# Phosphate-Solubilizing Bacteria Mobilize Nano-Hydroxyapatite In Vitro with Limited Transferability to Maize (*Zea mays* L.) Phosphorus Nutrition in Soil

**DOI:** 10.3390/plants15182869

**Published:** 2026-09-19

**Authors:** Theo Wilde, Johanna Marie Lass, Marcel Naumann

**Affiliations:** Division Plant Nutrition and Crop Physiology, Department of Crop Sciences, Georg-August-Universität Göttingen, Carl-Sprengel-Weg 1, 37075 Göttingen, Germanymarcel.naumann@agr.uni-goettingen.de (M.N.)

**Keywords:** phosphate mobilization, organic acid production, calcium phosphate dissolution, rhizobacteria, phosphorus deficiency, plant–microbe interactions, sustainable fertilization

## Abstract

Limited phosphate rock resources and inefficient phosphorus (P) fertilization challenge sustainable crop production. Nano-hydroxyapatite (nHA) serves as a model for poorly soluble calcium phosphates relevant to recycled P fertilizers. We tested whether phosphate-solubilizing bacteria (PSB) enhance P mobilization from nHA and maize (*Zea mays* L.) nutrition under moderate-temperature conditions. In vitro assays evaluated six bacterial strains and organic acid (OA)-mediated P mobilization; a climate-chamber pot experiment tested nHA, PSB, and their combinations under low and moderate P supply. Several strains increased supernatant P concentrations in vitro; *Pseudomonas rhodesiae* showed the strongest response, corresponding to 90% of the P introduced with nHA. OA-mediated P mobilization was greater at pH 3.5 than at pH 5.0, although different OA concentrations were used to reach the respective target pH values. The descriptive differences among OAs may reflect additional OA-specific interactions with Ca, including complexation. In the pot experiment, shoot P uptake did not differ significantly among treatments under low P supply. Under moderate P supply, the treatment with nHA depot fertilization without added medium or bacterial inoculation showed the numerically highest shoot P uptake and dry matter production. No consistent additional PSB-mediated benefit relative to the corresponding sterile-medium controls was demonstrated. Strong in vitro nHA mobilization showed limited transferability to buffered soil systems.

## 1. Introduction

Phosphorus (P) is an essential macronutrient and a central determinant of stable and high crop yields in modern agriculture [1,2]. Its importance derives from its fundamental involvement in key physiological processes in plants [3] as well as in the surrounding ecosystem in soil [4]. At the cellular level, P is an integral component of nucleic acids and phospholipids, contributes to membrane integrity and genetic information transfer, and plays a key role in energy metabolism as part of adenosine triphosphate and adenosine diphosphate. In addition, inorganic P is involved in enzymatic regulation, phosphorylation reactions, signal transduction, and photosynthesis [5,6].

Despite its essentiality, P availability in agricultural systems is often limited. Even where total soil P levels are high, only a small proportion is directly accessible to plants [7]. Merely 5–30% of applied P fertilizer is taken up by crops during the first growing season [8]. This low efficiency mainly results from the rapid fixation of soluble P into sparingly available forms, particularly calcium (Ca) phosphates in neutral to alkaline soils [9]. To compensate for anticipated fixation, P fertilizers are often applied in excess, resulting in low P use efficiency [10]. Surplus P is prone to losses via surface runoff, contributing to eutrophication of aquatic ecosystems and associated environmental issues [11,12]. Compounding these challenges is the reliance of global agriculture on finite phosphate rock resources, primarily apatite deposits [13]. Due to finite reserves, geopolitical risks, environmental impacts, and P losses, a transition to circular P recycling is essential for long-term P security [14]. Together, these factors define a central challenge in contemporary agriculture: the excessive use of a finite P resource to overcome rapid soil fixation, ultimately causing inefficient P use and substantial environmental impacts.

Hydroxyapatite (HA; Ca_5_(PO_4_)_3_(OH)) has emerged as a promising carrier for recycled P [15]. Its high P content (~18.5%) and notable thermal stability [16] make it particularly suitable for processing, including hygienization steps that reduce organic contaminants such as pathogens or residual pharmaceuticals [17]. HA can be recovered from various secondary sources, including sewage sludge [18] and biogenic residues such as bones, fish skeletons, and crustacean shells [19,20]. Consequently, it represents a valuable option in circular nutrient management strategies aimed at conserving finite P resources. However, a major limitation of HA is its extremely low solubility in water, which reduces P release and subsequent fixation in soil, while simultaneously limiting plant P availability and thus the agronomic effectiveness of HA [21,22]. The dissolution of HA and the plant availability of P derived from HA are influenced by particle size [23]. Since smaller particles provide a larger reactive surface area, reducing HA to the nanoscale has been proposed as an incremental strategy to improve dissolution processes. Nano-hydroxyapatite (nHA), defined as particles smaller than 100 nm [24], exhibits a substantially increased specific surface area that may accelerate P release [25]. However, P release from HA remains dependent on multiple factors including soil pH, soil type, nHA origin, temperature, cropping system and cultivation practices. Consequently, nanostructuring alone does not reliably overcome the fundamental limitation of HA under field-relevant conditions, namely the insufficient and poorly predictable supply of plant-available P [26]. In the present study, synthetic nHA was used as a model compound for poorly soluble calcium phosphate phases. Similar phases may occur in P fertilizers recovered from secondary resources, although the composition, crystallinity, particle size distribution, and agronomic behavior of such recycled materials can differ substantially from those of synthetic nHA.

To access sparingly soluble P sources in soil, plants engage in interactions with microorganisms in natural systems [27]. Among these microorganisms, phosphate-solubilizing bacteria (PSB) can play a central role in mobilizing mineral-bound P and increasing its availability for plant uptake [19]. The primary mechanisms of PSB-mediated solubilization involve the excretion of organic acids (OAs) which lower the pH in the rhizosphere and thereby increase the solubility of HA [28]. In addition, OAs such as citrate and 2-ketogluconic acid can chelate Ca^2+^ ions, thereby helping to maintain dissolved P in solution [29].

Beyond these dominant pathways, several complementary microbial processes influence P availability, including enzymatic activity, proton release associated with microbial metabolism (e.g., nitrification), and the dynamic immobilization of P in microbial biomass followed by its potential remineralization during microbial turnover [30,31]. To exert localized control over their immediate microenvironment and thereby enhance their solubilization capacity, PSB produce extracellular polymeric substances, which facilitate biofilm formation. The high specific surface area of nanoparticles provides abundant sites for biofilm development, enabling the localized accumulation of OAs directly at the nanoparticle interface [32,33]. Experimental evidence supports the effectiveness of such biological mobilization processes. For instance, PSB have been shown to release up to 40% of bound P from biogenic materials under in vitro conditions, while also mobilizing poorly soluble mineral phosphates and other sparingly available P forms in soil [34,35,36]. Combining nHA with PSB may enhance localized P mobilization. However, the magnitude and timing of this effect are expected to depend strongly on microbial establishment, soil buffering, fertilizer placement, and environmental conditions. Accordingly, the present study used a targeted set of six taxonomically diverse bacterial strains with literature-supported phosphate-solubilizing properties as candidates for comparative screening of nHA mobilization under standardized in vitro conditions.

Most current knowledge on P solubilization processes is derived from laboratory incubations [19] and experiments conducted under idealized conditions [37]. While these approaches provide valuable mechanistic insights, their transferability to agricultural systems remains limited due to simplified environmental settings. In particular, studies integrating practice-relevant temperature regimes and realistic fertilizer placement remain scarce, although both strongly influence microbial activity and P mobilization in soil [38]. This limitation is especially relevant in temperate agricultural systems, where crops are often exposed to comparatively low temperatures during early establishment. Such conditions can constrain microbial activity and enzyme-mediated nutrient cycling processes [39], thereby limiting PSB-mediated P mobilization. Climate chamber experiments simulating these moderate conditions provide a suitable intermediate step between laboratory studies and field trials. A particularly relevant crop in this context is maize (*Zea mays* L.), which is characterized by high early-season P demand and strong responsiveness to P availability [3,40]. Accordingly, localized starter fertilization (depot fertilization) and under-root fertilizer placement are widely used to improve early P supply. In parallel, advances in fertilization and inoculation technologies, including in-furrow application systems that place microbial inoculants directly near the seed and emerging roots, increasingly enable the targeted co-application of fertilizers and microbial inoculants under agronomically realistic conditions [41,42]. Maize was therefore selected as an agronomically relevant test crop because its early P sensitivity, cultivation under moderate early-season temperatures, and common use of P depot fertilization provide a suitable system for evaluating whether PSB-mediated nHA mobilization can improve plant P acquisition under soil-based conditions.

Against this background, the present study investigated whether the strong P-mobilization potential of PSB observed under controlled in vitro conditions could be translated into improved P availability, P uptake, and plant growth when combined with nHA under soil-based, moderate-temperature conditions representative of early-season maize growth in Central Europe. It was hypothesized that (i) selected PSB would increase P concentrations in the supernatant fraction of nHA-containing cultures; (ii) nHA-containing depot fertilization treatments would increase soil P availability, with PSB inoculation providing an additional benefit under moderate-temperature conditions; and (iii) nHA-containing depot fertilization treatments would increase P uptake and plant growth, with PSB inoculation providing an additional benefit.

## 2. Materials and Methods

The study comprised three experiments. Two in vitro experiments were first conducted to select the PSB based on their solubilization capacity and to investigate potential mechanisms of nHA dissolution. Subsequently, a soil-based climate chamber pot experiment with maize was conducted.

### 2.1. In Vitro Selection of Bacterial Strains Based on Phosphate-Solubilizing Activity

The six bacterial strains listed in Table 1 were preselected as candidate PSB based on literature reporting phosphate-solubilizing properties and potential relevance for plant P acquisition. All strains were represented by DSMZ accession numbers and were available either as dried cultures or as laboratory glycerol stocks. DSM 21393, DSM 6125, and DSM 30034 had additionally been used in previous crop–PSB experiments within our research context, whereas DSM 100941, DSM 7527, and DSM 17221 were included as literature-supported candidates to broaden the taxonomic range of the screening. The purpose of the initial in vitro experiment was therefore not to provide an exhaustive comparison of PSB diversity, but to screen these preselected candidates for their ability to mobilize P from nHA under standardized conditions. Strains were obtained from dried cultures or glycerol stocks and pre-cultivated in their respective growth media under strain-specific conditions. For the establishment of main cultures, pre-cultivated strains were transferred to strain-specific liquid media and incubated at 27 °C and 140 rpm until reaching exponential growth. Cell densities were standardized to an optical density at 600 nm (OD_600_) of 0.1 using P-free medium, corrected for medium-specific background absorbance and measured within the linear measurement range (0.1–1.2) of OD_600_-based quantification. Cells were not washed before inoculation, resulting in a small residual carryover of the respective strain-specific preculture medium. Colony-forming units (CFU) were determined by serial dilution [43] and plate colony counting using the Cell Counter plugin in ImageJ 1.54p (National Institutes of Health, Bethesda, MD, USA). For P solubilization assays, a P-free bacterial medium was prepared using the National Botanical Research Institute’s phosphate growth medium (NBRIP; [44]). As the only P source, nHA (<65 nm particle size, ≥97%, synthetic) was added at a concentration of 0.8 g L^−1^, ensuring the formation of a homogeneous suspension. Aliquots of 50 mL of the autoclaved NBRIP + nHA suspension were dispensed into Erlenmeyer flasks. A 100 µL aliquot of each OD-standardized culture was used to inoculate the prepared medium. Uninoculated controls containing the same autoclaved NBRIP + nHA suspension without bacterial addition were incubated and sampled in parallel. Three independently inoculated flasks were prepared per bacterial strain and for the uninoculated control. The flasks were incubated at 27 °C with horizontal shaking at 140 rpm. The same flasks were repeatedly sampled after 24 h, 2 days, 5 days, and 7 days of incubation, and the collected samples were immediately frozen to stop biological activity. Frozen samples were thawed and centrifuged at 9168× *g* for 10 min. The aqueous supernatant was then separated from the pellet containing bacterial cell residues and residual nHA particles and stored at −20 °C until analysis.

### 2.2. In Vitro Assessment of OA-Mediated P Solubilization from nHA

To obtain initial insights into metabolite production associated with phosphate solubilization, a metabolite screening analysis was performed on four bacterial strains selected based on their performance in the preceding nHA solubilization experiment (Table 2). Due to the exploratory screening character of this analysis, no biological replication was included and the resulting metabolite profiles were interpreted qualitatively rather than quantitatively. Culture supernatants were analysed by nuclear magnetic resonance spectroscopy by a commercial service provider (Lifespin GmbH, Regensburg, Germany). The results were used to identify predominant OAs and served as a basis for the design of the subsequent experiment investigating OA-mediated P mobilization from nHA.

A follow-up experiment was designed to investigate the role of OAs in the solubilization of nHA under controlled conditions. Because proton activity strongly affects nHA dissolution [53], a pH-based approach was used to compare OA-specific P solubilization at defined pH endpoints. Additional OAs reported to contribute to P solubilization include gluconic, oxalic, and propionic acid [54,55,56]. Stock solutions (0.5 M) of each OA were prepared and added stepwise to the autoclaved NBRIP + nHA suspension at an nHA concentration of 0.8 g L^−1^. The pH of each suspension was measured using a pH meter (Docu-pH Meter, Sartorius, Göttingen, Germany) and adjusted to defined target values of pH 5.0 and pH 3.5 by stepwise addition of the respective acid, followed by equilibration to ensure stable conditions. The volume of acid required to reach the target pH was recorded for each treatment (Table A1). For each OA × pH combination, one suspension was prepared, adjusted to the target pH, homogenized, and subsequently divided into three 2 mL microcentrifuge tubes. The tubes were incubated separately at 27 °C with shaking for 48 h. Because each treatment suspension was prepared only once before aliquoting, the three tubes were considered technical incubation replicates rather than independently prepared experimental replicates. Control samples without added OAs were included. After incubation, samples were centrifuged at 9168× *g* for 10 min to separate residual nHA from the liquid phase, and the aqueous supernatant was collected and stored at −20 °C until further analysis.

To facilitate a descriptive comparison of P mobilization across OAs and pH levels, P solubilization efficiency (PSE) was calculated. PSE was defined as the ratio of supernatant P concentration (mg P L^−1^) to the amount of OA applied (mmol L^−1^), resulting in a unit of mg P mmol OA^−1^. PSE was used to normalize P mobilization to the amount of OA added but was not interpreted as an intrinsic OA-specific efficiency because the acid amounts required to reach the target pH differed markedly among OAs. The calculated PSE values were therefore evaluated descriptively.

$$PSE=\frac{Supernatant\;P\;}{Applied\;OA}$$

### 2.3. Determination of P and Ca in Bacterial and OA Solubilization Experiments

The analysis was performed analogously for samples derived from both the bacterial solubilization experiments and the OA-mediated solubilization assays. Frozen samples were thawed and centrifuged at 9168× *g* for 3 min to remove residual particles. Complete removal of nanoscale or colloidal nHA from the supernatant was not independently verified. Subsequently, 1 mL of the supernatant was diluted 1:10 in 5% nitric acid as the measurement matrix. Samples were analysed using inductively coupled plasma optical emission spectrometry (ICP-OES; Genesis FES, Spectro, Kleve, Germany) to determine total P and Ca concentrations in the supernatant.

### 2.4. Pot Experiment with Maize Using Combined Application of PSB and nHA

#### 2.4.1. Experimental Design and Treatments

The pot experiment followed a factorial design with two P fertilization levels and six treatment variants, resulting in 12 treatment combinations with six replicates each (*n* = 6), totalling 72 pots. Two P supply levels were established (Table 3): (i) a low P supply level, corresponding to the initial CAL-extractable soil P content of 10.7 mg P kg^−1^ soil (CAL method A 6.2.2.1, [57]) without additional P fertilization, and (ii) a moderate P supply level, achieved by P amendment to reach a target P level of 40 mg P kg^−1^ soil. This level was selected to ensure sufficient P supply for plant growth while still allowing the detection of additional fertilization effects of nHA. P was applied as disodium hydrogen phosphate dihydrate (Na_2_HPO_4_ ∙ 2H_2_O). Six treatment variants were established within each P level: (i) control, (ii) sterile medium (+sM), (iii) bacterial inoculation (+B), (iv) nHA depots (+nHA), (v) sterile medium combined with nHA (+nHA+sM), and (vi) PSB inoculation combined with nHA (+nHA+B).

#### 2.4.2. Soil Preparation and Planting

**Figure A1:**
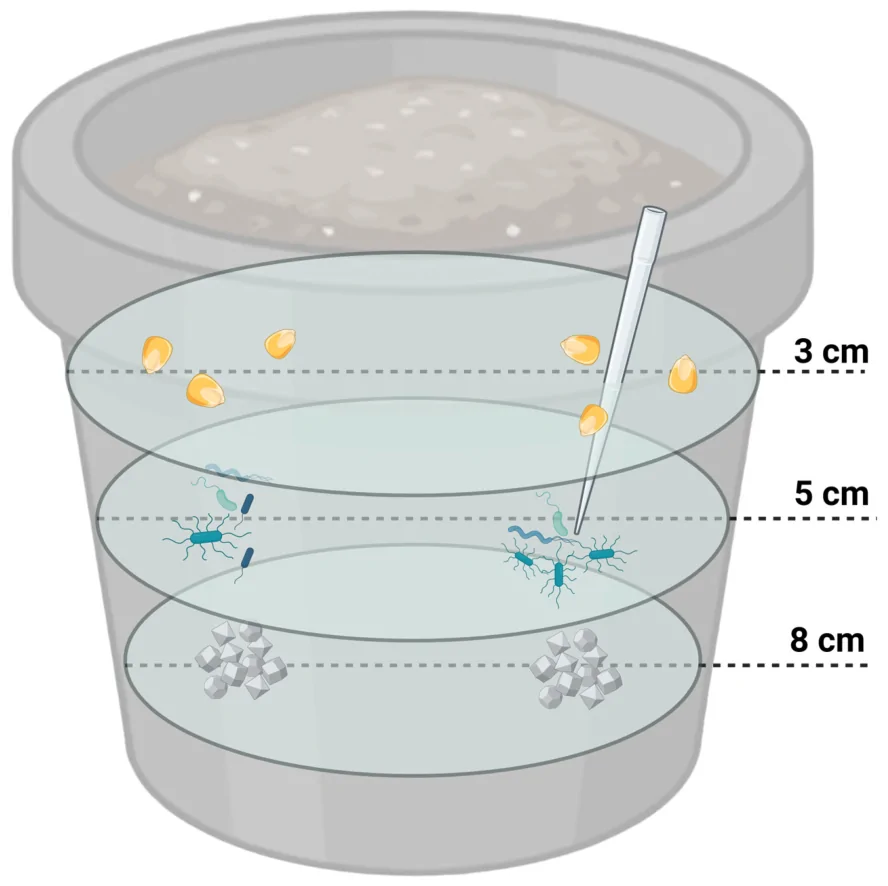
Schematic illustration of the pot experiment setup used in the climate chamber. Maize seeds were sown at a depth of 3 cm, while bacterial inoculum and nHA were locally placed at depths of 5 cm and 8 cm, respectively. The spatial separation was designed to enable root-mediated interaction with the bacterial inoculum and locally placed nHA during plant development. An approximately 1.5 cm quartz sand layer was applied to the soil surface during experimental setup. Created in BioRender. Wilde, T. (2026) https://BioRender.com/dd6t736.

Pots were filled with 2.5 kg of topsoil as substrate. The soil was collected from the Leine Valley near Göttingen, Germany (51°27′43.7″ N, 10°08′47.4″ E), air-dried, and passed through a 5 mm sieve before use. According to the World Reference Base for Soil Resources [58], the soil was classified as a deep, eroded Regosol (Loamic) developed from silicate weathering material [59]. The soil texture was classified as a weakly clayey loam using the texture-by-feel method (LUFA Nord-West, Hameln, Germany). Prior to the experiment, soil P and K concentrations were determined by LUFA Nord-West (Hameln, Germany) using calcium acetate lactate (CAL) extraction according to VDLUFA method A 6.2.2.1 [57], while Mg was determined using CaCl_2_ extraction. Soil pH was measured potentiometrically using a pH electrode in a CaCl_2_ solution (A 5.1.1, [57]). Soil mineral N (NH_4_^+^-N and NO_3_^−^-N) was extracted by shaking the soil samples in 0.0125 M CaCl_2_ for 1 h. The extracts were filtered using MN 615 ^1^/_4_ filter paper (Macherey-Nagel, Düren, Germany) and analyzed with a continuous flow analyzer (Skalar Analytical, Breda, The Netherlands). The results of the pre-experimental soil analysis, including the initial nutrient concentrations and soil pH, are summarized in Table 3. The soil was selected because of its low initial CAL-extractable P concentration of 10.7 mg P kg^−1^ soil. Based on the pre-experimental soil analyses, all treatments received a uniform basal nutrient supply to avoid limitations by nutrients other than P. For nutrients with measured initial concentrations, fertilizer additions were calculated to achieve the target concentrations shown in Table 3. Potassium and magnesium were supplied as potassium sulfate (K_2_SO_4_) and magnesium sulfate heptahydrate (MgSO_4_ · 7H_2_O). Nitrogen was supplied as ammonium nitrate (NH_4_NO_3_) and Ca nitrate tetrahydrate (Ca(NO_3_)_2_ · 4H_2_O). Lime was applied as Ca carbonate (CaCO_3_) according to standard liming recommendations [60] to maintain physiologically suitable soil pH under pot experimental conditions with high plant density relative to the limited soil volume. For treatments including nHA, nHA was applied at two localized sites (depots) per pot at a depth of 8 cm, one beneath each final plant position. A total of 0.2 g nHA was applied per pot, corresponding to 14.8 mg P kg^−1^ soil, representing a moderate P supply rate relevant to field conditions (Table 3). To ensure homogeneous distribution and prevent nanoparticle aggregation, the nHA was mixed with 3 g of air-dried soil sieved to <0.5 mm. In all nHA-containing treatments (+nHA, +nHA+sM, and +nHA+B), 0.5 g sucrose per pot was additionally included as a readily available carbon and energy source to support microbial activity alongside P mobilization. The nHA-containing mixture was placed at two designated locations within each pot at a depth of 8 cm (Figure A1).

Six maize seeds (*Zea mays* L., cultivar KWS Emporio, 2025, untreated seeds) were subsequently sown at a depth of 3 cm in two groups of three seeds, with each group positioned above one nHA fertilization depot. Following sowing, bacterial inoculation was performed using a four-strain consortium comprising DSM 7527, DSM 17221, DSM 6125, and DSM 100941, which had been selected based on their comparatively high P concentrations in the supernatant fraction in the preceding in vitro screening. To achieve comparable contributions of each strain within the consortium, all cultures were adjusted to the same optical density (OD_600_ = 0.32). This value was defined by the strain reaching the lowest maximum density, while denser cultures were diluted with P-free medium accordingly. For inoculation, two holes (5 cm depth) were created per pot above the localized nHA fertilizer depots and filled with 5 mL of bacterial consortium suspension each (total 10 mL pot^−1^). The holes were subsequently closed with soil (Schematic illustration: Figure A1). A layer (~1.5 cm) of quartz sand was applied to the soil surface to reduce evaporation and standardize pot weight. After emergence, plants were thinned at BBCH 12 by reducing at both positions from three to one plant, resulting in two plants per pot, one at each of the two designated plant positions.

The experiment was conducted in a climate chamber (Fitotron SGR221, Weiss Technik, Loughborough, UK) under a randomized design, with pots re-randomized halfway through the experiment to minimize positional effects. Conditions simulated early-season maize growth in Central Europe. Day/night conditions were set to 18/14 °C and 60/80% relative humidity. A photoperiod of approximately 13.5 h with dynamic light control and a light intensity of 218 µmol m^−2^ s^−1^ photosynthetic photon flux density (LI-180 spectrometer, LI-COR, Lincoln, NE, USA) was applied. Soil moisture was maintained at approximately 60% of field capacity using a drip irrigation system with deionized water.

#### 2.4.3. Analysis of the Pot Experiment

Plant performance was assessed non-destructively before harvest by measuring shoot length and visually documenting nutrient deficiency symptoms. Plants were harvested 51 days after planting. Shoots were cut directly at the soil surface and collected from all replicates (*n* = 6). Roots were collected from a subset of three replicates per treatment (*n* = 3) and washed to remove adhering soil particles. Shoot and root samples were dried at 60 °C to constant weight and subsequently weighed to determine dry matter (DM). The dried plant material was then finely ground to 2 mm for subsequent chemical analysis. Ground plant material was subsequently subjected to microwave digestion (Ethos Terminal 660, MLS Mikrowellen-Labor-Systeme GmbH, Leutkirch im Allgäu, Germany) at 200 °C and 15 bar for 80 min using nitric acid (HNO_3_) and hydrogen peroxide (H_2_O_2_). The resulting digests were analysed for elemental concentrations using ICP-OES. After harvest, soil from each pot was collected and homogenized, and CAT-extractable P was determined using CaCl_2_ and DTPA according to method A 6.4.1 [57], followed by ICP-OES analysis. Soil pH was measured potentiometrically using a pH meter in a CaCl_2_ solution (A 5.1.1, [57]).

The apparent shoot P recovery efficiency associated with nHA application (PRE_nHA_) was calculated using a difference method adapted from Johnston and Syers [61]. PRE_nHA_ was defined as the difference in shoot P uptake between corresponding treatments with and without nHA, divided by the amount of P applied as nHA and expressed as a percentage:

$${PRE}_{nHA}=\frac{\left({P\;uptake}_{+nHA}\right)-({P\;uptake}_{-nHA})}{{P\;applied}_{nHA}}\times 100$$

P uptake_+nHA_ represented the shoot P uptake of maize in the respective treatment receiving nHA, and P uptake_-nHA_ represented the shoot P uptake in the corresponding treatment without nHA. P applied_nHA_ corresponded to the amount of P applied via nHA (37 mg P pot^−1^). As PRE_nHA_ was derived from differences in shoot P uptake rather than from isotope-labelled P, it represented an apparent treatment-associated recovery and did not provide source-specific evidence that the additionally acquired P originated directly from nHA. Shoot P uptake was calculated as the product of shoot DM and shoot P concentration. Root P uptake was not included in the recovery calculations because root biomass was assessed only for a subset of replicates (*n* = 3), whereas shoot biomass was available for all replicates (*n* = 6). In addition, root-based estimates are more susceptible to uncertainty associated with adhering soil particles and biomass losses during root washing. The calculation was performed using corresponding treatment pairs to isolate the effect of nHA: control vs. +nHA, +sM vs. +nHA+sM, and +B vs. +nHA+B. To further isolate the contribution of PSB activity, an adjusted recovery efficiency (PRE_B_) was calculated by modifying the comparison used for PRE_nHA_. Instead of comparing treatments with and without nHA, the effect of living PSB was assessed within the nHA treatments by comparing the treatment receiving both nHA and bacteria (+nHA+B) with the corresponding sterile medium control (+nHA+sM).

### 2.5. Statistical Analysis

Statistical analyses were performed separately for each experiment and response variable. For the bacterial nHA-solubilization experiment, the association between all paired supernatant P and Ca concentrations across time was additionally characterized using Pearson’s correlation coefficient. The OA-mediated nHA-mobilization experiment was evaluated descriptively. Means and standard deviations represented variation among three technical incubation replicates obtained from one independently prepared suspension per OA × pH combination. All remaining inferential analyses were based on one-way ANOVA within the relevant sampling day or P supply level. Normality of model residuals and homogeneity of variances were assessed using the Shapiro–Wilk and Levene’s tests, respectively. When assumptions were met, Tukey-adjusted comparisons of estimated marginal means were performed; when assumptions were violated, omnibus tests and post hoc comparisons were based on HC3 heteroscedasticity-consistent covariance estimates with Holm adjustment. Shoot DM was analysed separately within each P supply level, whereas root DM and the root-to-shoot ratio (R:S) were evaluated descriptively; R:S was calculated from matched root and shoot measurements of the same pots. PRE_nHA_ and PRE_B_ were estimated using model-based contrasts between independent treatment means. For these contrasts, *p*-values were adjusted using the Holm method, whereas the graphical error bars represented unadjusted pointwise 95% confidence intervals derived from the fitted models. Unless stated otherwise, data are presented as mean ± standard deviation (SD), and groups sharing at least one letter did not differ significantly at an adjusted *p* < 0.05. Statistical analyses were conducted in R version 4.3.2 (R Core Team, Vienna, Austria) using RStudio version 2026.01.1 and the packages *tidyverse*, *car*, *emmeans*, *multcomp*, *multcompView*, and *sandwich*.

## 3. Results

### 3.1. In Vitro Bacterial Solubilization of nHA

During the nHA solubilization experiment, visible strain-dependent differences in turbidity were observed in the nHA-amended suspension. DSM 21393 and DSM 30034 maintained consistently low turbidity throughout incubation, whereas the remaining strains showed substantially higher turbidity accompanied by a visible reduction in suspended nHA particles. As nHA was applied as a suspension, minor variation in particle distribution between replicates could not be fully excluded despite homogenization. P and Ca concentrations in the supernatant fraction were therefore additionally expressed relative to the theoretical total amounts introduced via nHA (148 mg P L^−1^; 319.2 mg Ca L^−1^) and should be interpreted comparatively rather than as absolute recovery values. The measured concentrations represent non-cumulative snapshots of P and Ca concentrations in the supernatant fraction over 7 days. The uninoculated NBRIP + nHA control contained 1–3 mg P L^−1^.

**Figure 1:**
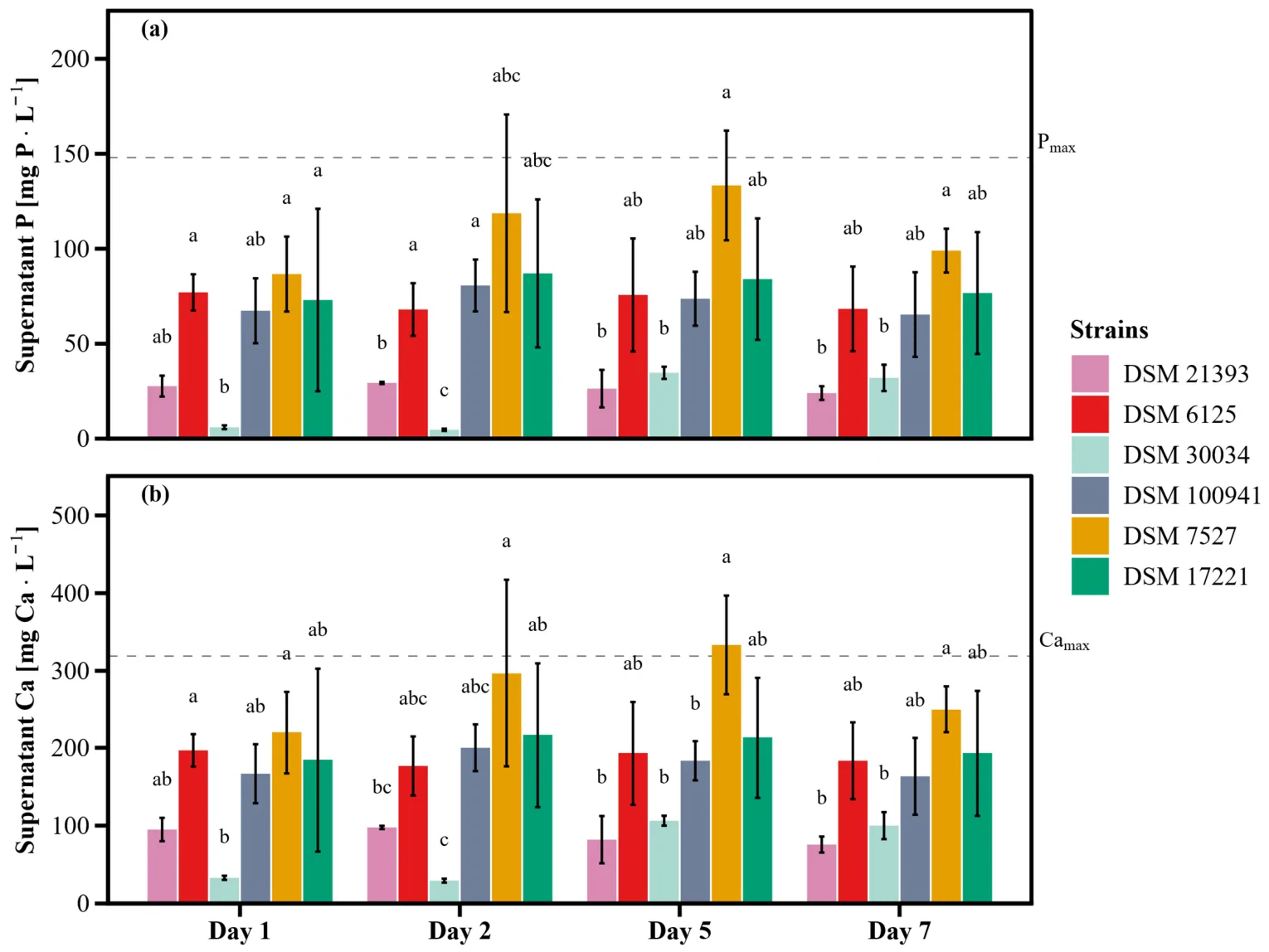
(**a**) Supernatant phosphorus (mg P L^−1^) and (**b**) calcium (mg Ca L^−1^) from nano-hydroxyapatite (nHA) due to phosphate-solubilizing bacteria (PSB) cultures over time. Measured for PSB strains DSM 21393, DSM 6125, DSM 30034, DSM 100941, DSM 7527 and DSM 17221 at one, two, five and seven days after incubation at 27 °C in liquid suspension of P-free medium with nHA. The theoretical maximum line (P_max_; Ca_max_) denotes the theoretical maximum of P (148 mg P L^−1^) or Ca (319 mg Ca L^−1^) mixed in the P-free medium + nHA suspension. Bars represent means and error bars indicate ±SD (*n* = 3). Letters indicate significant differences among PSB strains within the same day based on a one-way ANOVA followed by post hoc Tukey’s HSD test (*p* < 0.05).

Across all sampling times and bacterial treatments, supernatant P and Ca concentrations showed a close positive association (Pearson’s r = 0.997, 72 paired observations). Given the repeated sampling of the same flasks over time, this coefficient was used descriptively. While the calculated Ca:P ratio of the applied nHA was 2.16, a higher average ratio of 2.98 was measured in the supernatants. DSM 30034 showed the lowest P solubilization at the beginning (3.2% at day 2), while already releasing a higher proportion of Ca (9.2%). As shown in Figure 1, DSM 7527 exhibited the highest supernatant P concentrations, with the highest observed mean concentration of 133 ± 28.9 mg P L^−1^ on day 5, corresponding to 90.1% of the theoretical total P (P_max_). This value was significantly higher than those of DSM 21393 and DSM 30034. Other high-performing strains included DSM 17221 (peak: 87 ± 39 mg P L^−1^), DSM 100941 (peak: 80.7 ± 13.7 mg P L^−1^), and DSM 6125 (peak: 77 ± 9.5 mg P L^−1^), with no significant differences among these strains. In contrast, DSM 21393 and DSM 30034 showed consistently lower solubilization, with their highest observed mean supernatant P concentrations reaching only 29.3 ± 0.6 and 34.7 ± 3.2 mg P L^−1^, respectively, corresponding to less than 25% of P_max_.

Observed supernatant P concentrations varied across sampling days and strains (Figure 1). Several strains had their highest observed mean concentrations on day 5, whereas DSM 7527 showed similarly high observed values on days 2 and 5. Ca:P ratios also showed variation across sampling days. No statistical comparisons among sampling days were performed; therefore, these temporal patterns are reported descriptively only. Based on their overall P-solubilization performance under the applied OD-standardized assay conditions, DSM 7527, DSM 17221, DSM 6125, and DSM 100941 showed comparatively high supernatant P concentrations and were selected for further application as a consortium in the maize pot experiment.

### 3.2. In Vitro OA Solubilization of nHA

**Figure 2:**
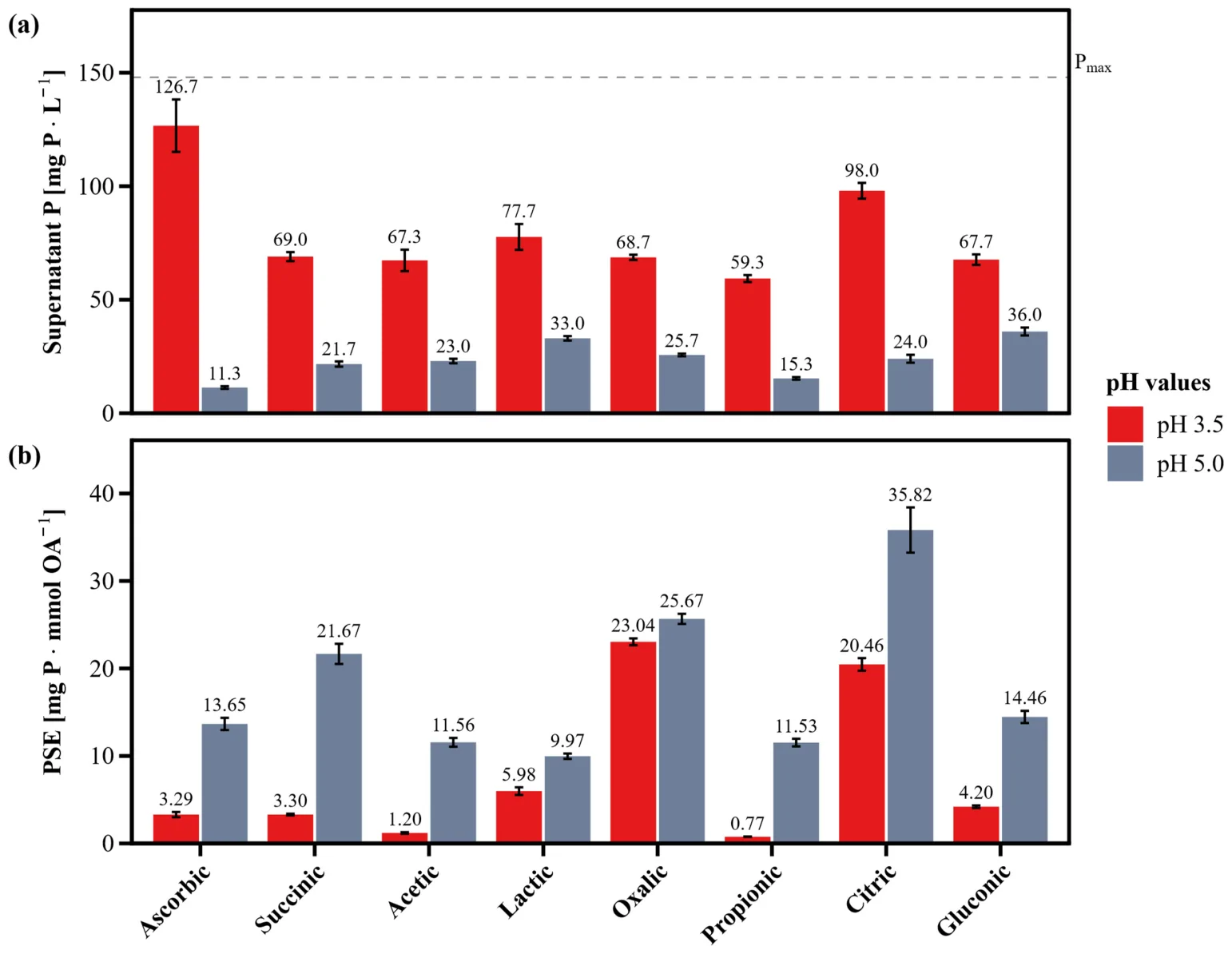
(**a**) Phosphorus concentration in the supernatant fraction (mg P L^−1^) and (**b**) calculated phosphorus solubilization efficiency (PSE; mg P mmol OA^−1^) following the addition of organic acids (OAs) to a liquid suspension of P-free medium containing nano-hydroxyapatite (nHA). Target pH values of 3.5 and 5.0 were established by stepwise addition of 0.5 M ascorbic, succinic, acetic, lactic, oxalic, propionic, citric, or gluconic acid. The theoretical maximum line (Pmax) denotes the total P concentration of 148 mg P L^−1^ introduced with nHA. Bars represent means, and error bars indicate ±SD of three technical incubation replicates obtained from one independently prepared suspension per OA × pH combination. Results were evaluated descriptively, and no inferential comparisons were performed.

All tested OAs resulted in measurable P concentrations in the supernatant fraction. Across OAs, the observed mean supernatant P concentrations were generally higher at pH 3.5 than at pH 5.0, with the descriptive difference averaging 37.5% of Pmax (Figure 2a). Consistent with this descriptive pattern, a more pronounced decrease in suspension turbidity was observed at pH 3.5 following OA addition. No inferential statistical comparisons between pH levels were performed because the replicates represented technical incubation replicates derived from a single independently prepared suspension per OA × pH combination. Descriptive differences were observed among the tested OAs. At pH 5.0, gluconic and lactic acid resulted in the highest supernatant P concentrations, reaching 36 ± 1.7 and 33 ± 1.0 mg P L^−1^, respectively, whereas propionic and ascorbic acid resulted in lower concentrations. At pH 3.5, the highest supernatant P concentration was observed for ascorbic acid at 126 ± 11.5 mg P L^−1^, followed by citric acid at 98 ± 3.5 mg P L^−1^. The remaining OAs resulted in concentrations ranging from 59.3 to 77.7 mg P L^−1^.

To account descriptively for differences in OA input, PSE was calculated (Figure 2b). For most OAs, calculated PSE values were higher at OA-adjusted pH 5.0 than at pH 3.5. Citric, oxalic, and succinic acid showed the highest calculated values at pH 5.0, reaching 35.8, 25.7, and 21.7 mg P mmol OA^−1^, respectively. Citric and oxalic acid also showed comparatively high PSE values at pH 3.5, whereas propionic and acetic acid showed low values at both pH levels.

### 3.3. Plant Growth and Biomass Production

**Figure 3:**
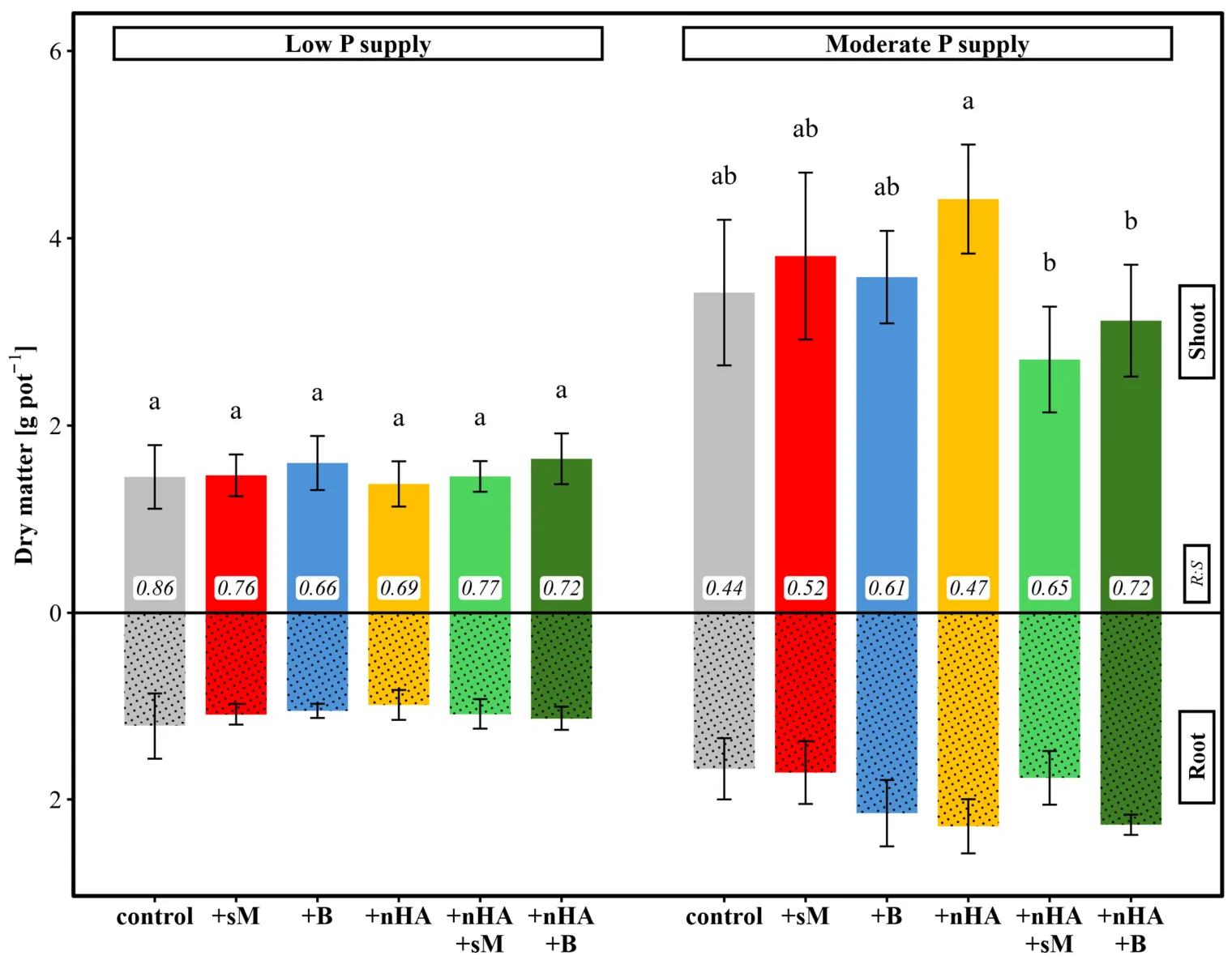
Shoot and root dry matter (DM; g pot^−1^) and root-to-shoot ratio (R:S) of maize (*Zea mays* L.) under low and moderate phosphorus (P) supply. Treatments were control, sterile medium (+sM), nano-hydroxyapatite (+nHA) and phosphate-solubilizing bacteria (+B) and their combinations. Values in white boxes show the R:S (*n* = 3), which is calculated as the ratio of root DM to shoot DM. Solid bars represent shoot DM, whereas dotted bars represent root DM. Bars represent means and error bars indicate ±SD (Shoot DM: *n* = 6; Root DM: *n* = 3). Different letters indicate significant differences among treatments within each P supply level for shoot DM according to one-way ANOVA followed by post hoc Tukey’s HSD test (*p* < 0.05).

**Figure A2:**
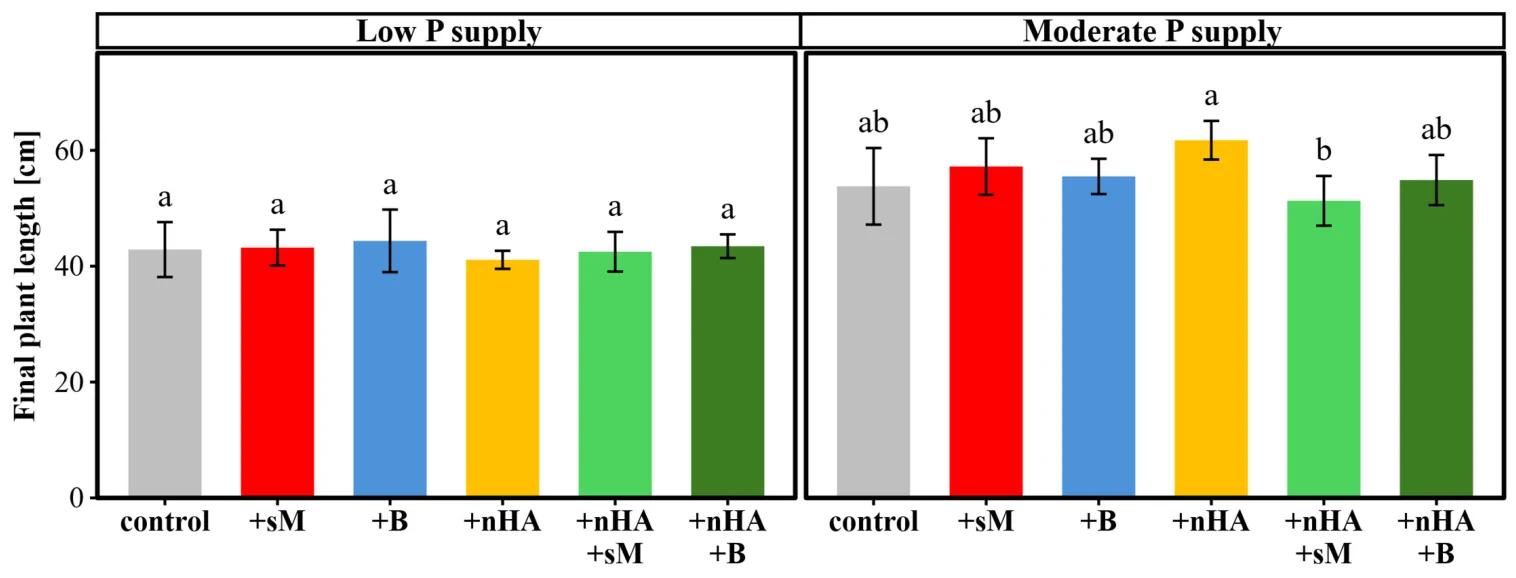
Final plant length (cm) of maize 51 days after planting under low and moderate phosphorus (P) supply. Treatments were control, sterile medium (+sM), nano-hydroxyapatite (+nHA), phosphate-solubilizing bacteria (+B), and their combinations. Bars represent means and error bars indicate ± SD (*n* = 6). Different letters indicate significant differences among treatments within each P supply level according to one-way ANOVA followed by Tukey’s HSD post hoc test (*p* < 0.05).

Shoot DM was affected by P supply, with plants under moderate P conditions producing on average 1.98 g higher shoot DM, corresponding to approximately 2.31-fold higher DM compared to low P supply (Figure 3). Within the moderate P level, the +nHA treatment showed the highest shoot DM (4.42 ± 0.58 g pot^−1^) and was significantly higher than +nHA+sM and +nHA+B, whereas no significant differences among treatments were observed under low P supply. Root DM followed a similar pattern, with generally higher values under moderate P supply. In contrast, the root-to-shoot ratio (R:S) was consistently higher under low P conditions and reached its maximum in the low-P control treatment (0.86). Plant length also differed markedly between P levels (Figure A2), with plants under moderate P supply being on average 12.8 cm taller. The greatest length was observed in the +nHA treatment under moderate P supply (61.8 ± 3.3 cm), whereas the +nHA treatment under low P supply resulted in the shortest plants (41.1 ± 1.6 cm). Despite these differences, plants across all treatments reached comparable developmental stages by harvest (51 days after planting), corresponding approximately to BBCH 15–16 under low P supply and BBCH 16–17 under moderate P supply.

**Figure A3:**
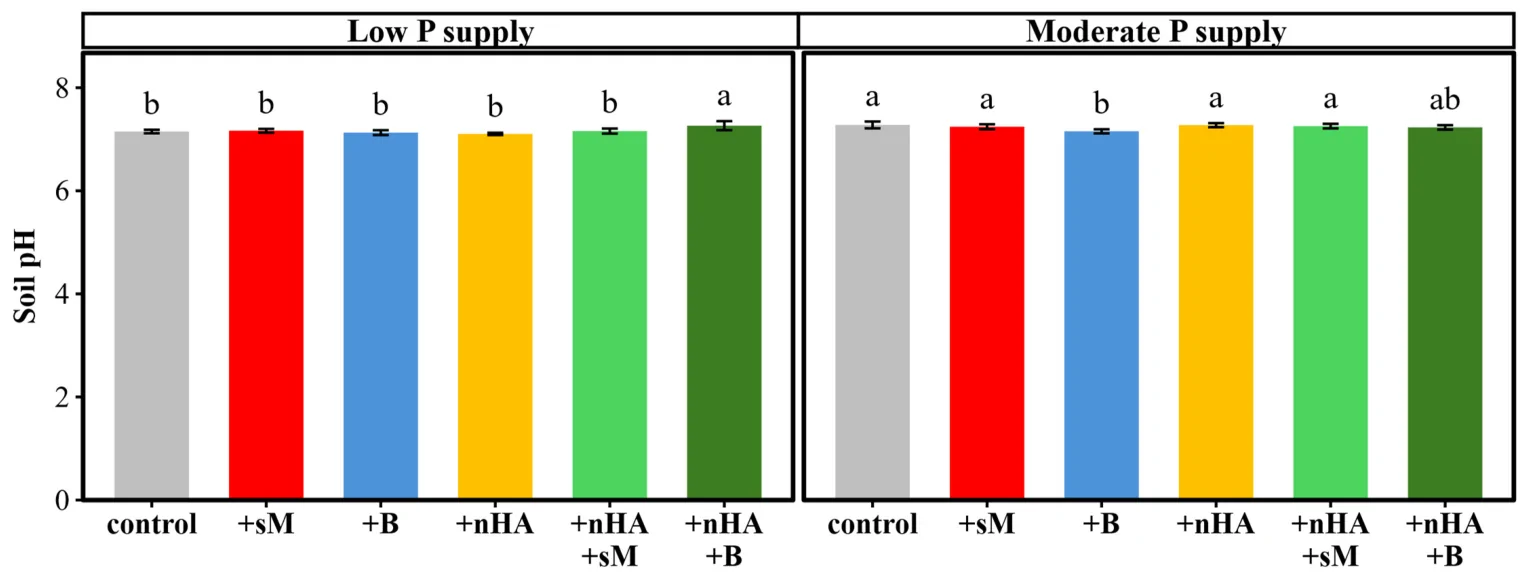
Soil pH after harvest under low and moderate phosphorus (P) supply in maize. Treatments were control, sterile medium (+sM), nano-hydroxyapatite (+nHA), phosphate-solubilizing bacteria (+B), and their combinations. Bars represent means and error bars indicate ± SD (*n* = 6). Different letters indicate significant differences among treatments within each P supply level according to one-way ANOVA followed by Tukey’s HSD post hoc test (*p* < 0.05).

These DM responses were consistent with visual observations during plant development. Shortly after emergence, plants developed clear P deficiency symptoms expressed as dark green to red-violet pigmentation of older leaves, accompanied by reduced growth and stunted development (Figure A3). Symptoms were consistently more evident under low P supply than under moderate P supply.

### 3.4. Maize P Acquisition and Soil Phosphorus Availability

**Figure 4:**
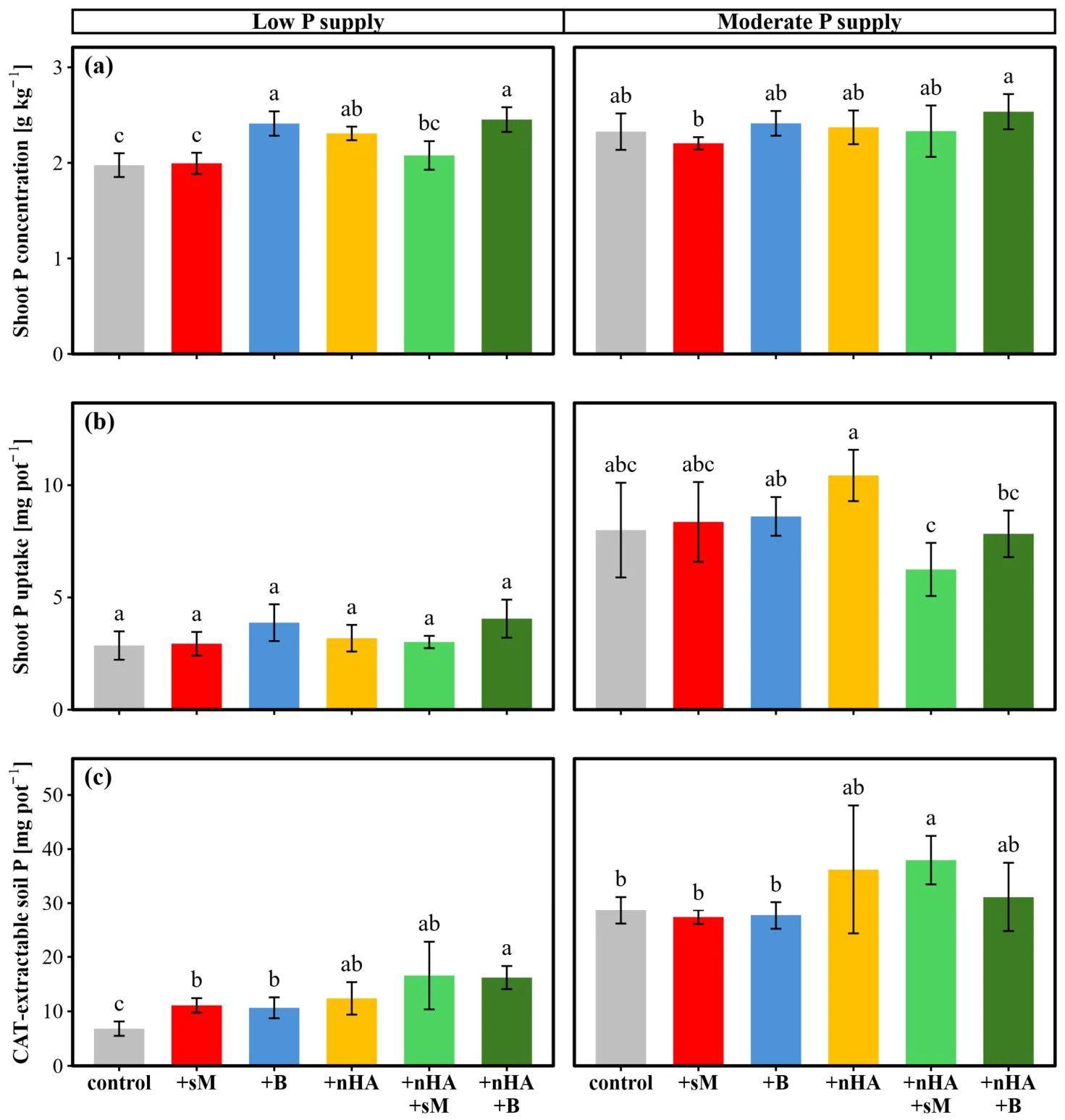
(**a**) Shoot phosphorus (P) concentration (g P kg^−1^), (**b**) shoot P uptake (mg P pot^−1^), and (**c**) CAT-extractable soil P (mg P pot^−1^) in maize under low and moderate P supply. Treatments were control, sterile medium (+sM), nano-hydroxyapatite (+nHA), phosphate-solubilizing bacteria (+B), and their combinations. Shoot P uptake was calculated from shoot dry matter and shoot P concentration. Bars represent means, and error bars indicate ±SD (*n* = 6). Different letters indicate significant differences among treatments within each parameter and P supply level according to the statistical procedures described in the Section 2.5.

The different P supply levels were also reflected in maize P acquisition (Figure 4a,b). Under low P supply, the highest shoot P concentration was observed for +nHA+B (2.49 ± 0.09 g P kg^−1^). The control and +sM treatments had significantly lower concentrations than +B, +nHA, and +nHA+B, whereas +nHA+sM was significantly lower than +B and +nHA+B but did not differ from +nHA. Under moderate P supply, +sM (2.20 ± 0.06 g P kg^−1^) had a significantly lower shoot P concentration than +nHA+B (2.54 ± 0.18 g P kg^−1^), which showed the highest value across all treatments and P supply levels. Shoot P uptake increased clearly with P supply, averaging 4.75 mg P pot^−1^ higher under moderate than under low P conditions. Under low P supply, no significant differences in shoot P uptake were detected among treatments, although +nHA+B showed the numerically highest value (4.20 ± 0.86 mg P pot^−1^). Under moderate P supply, +nHA resulted in the highest shoot P uptake (10.43 ± 1.14 mg P pot^−1^) and significantly exceeded +nHA+sM (6.25 ± 1.18 mg P pot^−1^) and +nHA+B (7.45 ± 0.52 mg P pot^−1^).

**Figure A4:**
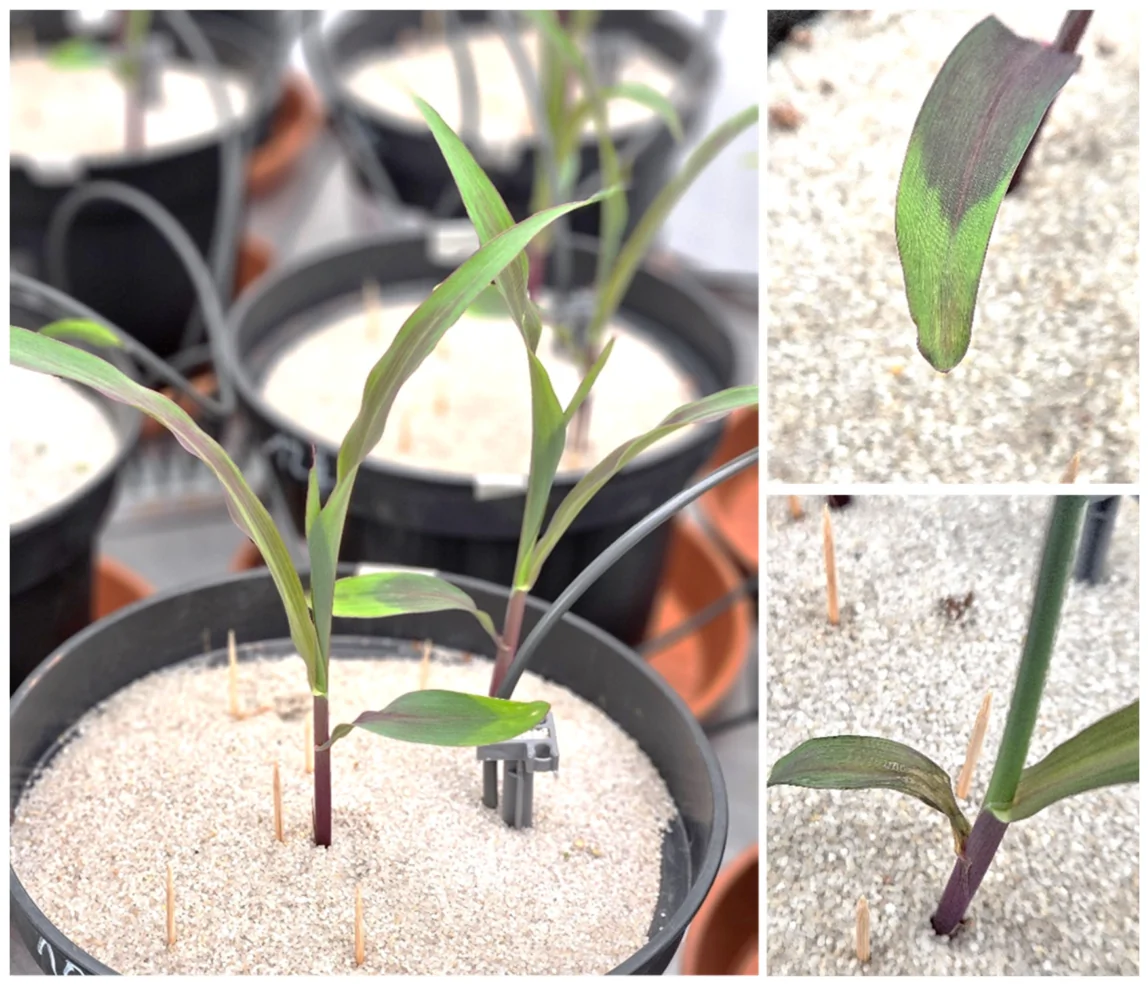
Representative phosphorus (P) deficiency symptoms observed in maize during the climate chamber experiment. After emergence, plants developed characteristic dark green to red-violet pigmentation of older leaves accompanied by reduced growth and stunted development under low P supply conditions. Photographs were taken 26 days after planting.

CAT-extractable soil P at harvest was also strongly affected by P supply (Figure 4c), averaging 19.9 mg P pot^−1^ higher under moderate than under low P conditions. Under low P supply, CAT-extractable soil P was lowest in the control (6.8 ± 1.3 mg P pot^−1^) and highest in the +nHA+B treatment (16.2 ± 2.1 mg P pot^−1^), which was significantly higher than in all treatments without nHA. Under moderate P supply, +nHA+sM showed the highest CAT-extractable soil P (39.4 ± 2.9 mg P pot^−1^) and was significantly higher than the control, +sM, and +B treatments, but did not differ significantly from +nHA or +nHA+B. Despite these differences in CAT-extractable P, soil pH remained within a narrow range around 7.2, although small treatment-dependent differences were detected (Figure A4).

#### Recovery Efficiency of Applied nHA-Phosphorus

**Figure 5:**
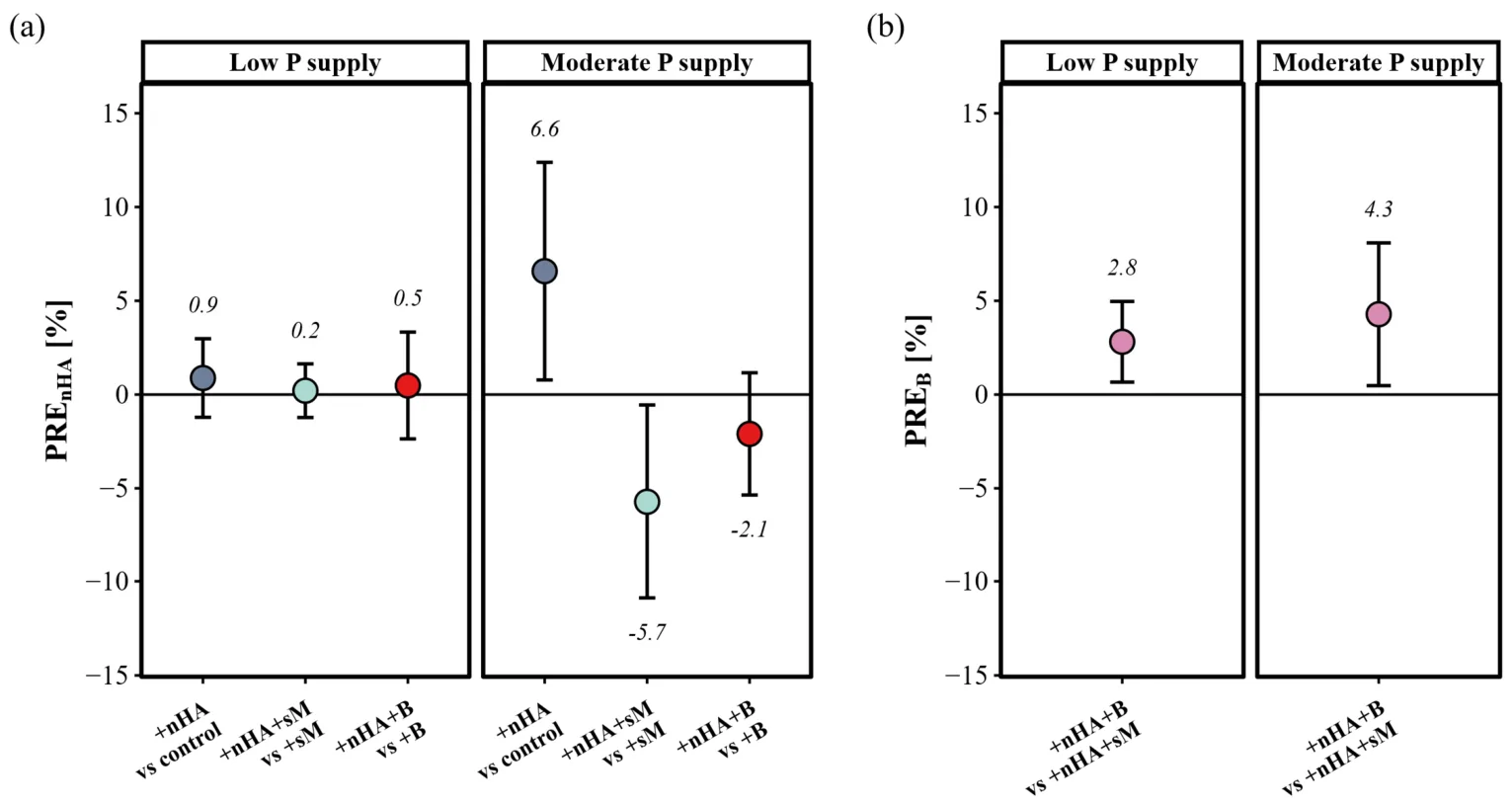
Model-based estimates of (**a**) apparent phosphorus recovery efficiency associated with nano-hydroxyapatite application (PRE_nHA_) and (**b**) apparent phosphorus recovery efficiency associated with living phosphate-solubilizing bacteria within nHA-containing treatments (PRE_B_) in the maize pot experiment. PREnHA was estimated from the contrasts +nHA minus control, +nHA+sM minus +sM, and +nHA+B minus +B within each P supply level. PREB was estimated from the contrast +nHA+B minus +nHA+sM within each P supply level. Points represent estimated contrasts, and error bars indicate unadjusted pointwise 95% confidence intervals. *p*-values for the contrasts were adjusted using the Holm method. The horizontal reference line at 0% indicates no difference between the respective treatments. Each treatment mean was based on six independent pots.

To further assess the apparent recovery associated with nHA application, phosphorus recovery efficiencies were calculated. Under low P supply, PRE_nHA_ point estimates remained below 1% and differed only slightly among +nHA, +nHA+sM, and +nHA+B (Figure 5a). Under moderate P supply, the PRE_nHA_ point estimate was highest for +nHA (6.6%) and was negative for +nHA+sM (−5.7%) and +nHA+B (−2.1%). Negative PRE_nHA_ values arise when shoot P uptake in an nHA-containing treatment is lower than in its corresponding non-nHA comparator. PRE_B_, representing the contrast between +nHA+B and +nHA+sM, was estimated at 2.8% under low and 4.3% under moderate P supply (Figure 5b).

## 4. Discussion

### 4.1. In Vitro P Mobilization from nHA by PSB and Their OAs

Hypothesis (i), proposing that selected PSB increase P concentrations in the supernatant fraction of nHA-containing cultures, was supported by the in vitro results. All tested strains resulted in elevated P concentrations in the supernatant fraction relative to the uninoculated control, consistent with phosphate-solubilizing activity. However, because complete removal of nanoscale or colloidal nHA particles from the supernatant was not independently verified, the measured P concentrations cannot be interpreted as representing exclusively dissolved P. The close association between supernatant Ca and P concentrations is consistent with the concurrent mobilization of both elements from nHA. (Figure 1). Elevated Ca:P ratios in the supernatant may reflect preferential microbial P uptake relative to Ca, as well as the commonly observed initial release of Ca during HA dissolution [62]. DSM 7527 temporarily reached P concentrations in the supernatant fraction corresponding to up to 90% of the theoretical maximum P in suspension, indicating that a high proportion of the applied P was detected in the supernatant fraction at the respective sampling time. The pronounced strain-specific differences further highlight PSB selection as a critical factor for agronomic applications, which could be further improved by including factors like temperature and pH conditions [63,64].

The qualitative metabolite screening showed that the selected strains produced several OAs potentially relevant to P mobilization, including succinate, acetate, ascorbate, citrate, and lactate (Table 2). However, because no biological replication was included, the data do not allow a quantitative relationship between OA production and strain-specific P mobilization to be established. In addition, the uniform NBRIP medium may not have equally met the nutrient requirements of the individual strains, potentially affecting both metabolite production and apparent nHA mobilization [65].

**Figure A5:**
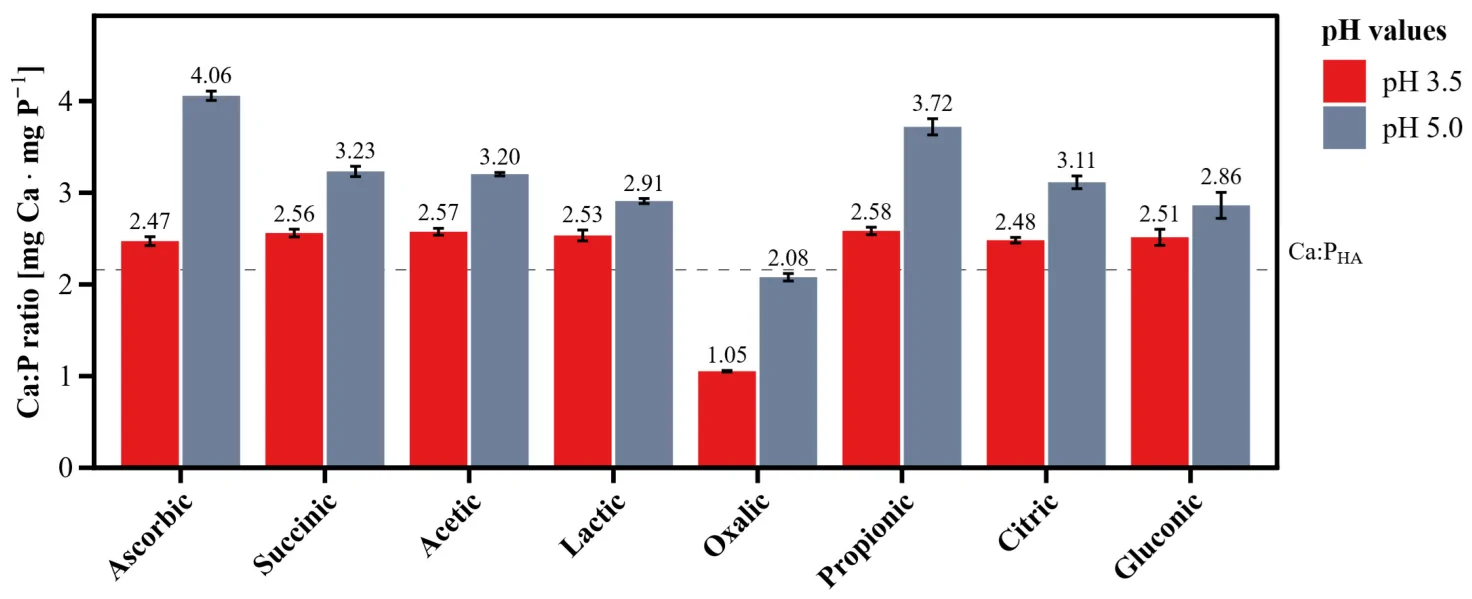
Ca:P ratio (mg Ca mg P^−1^) in the supernatant fraction of P-free medium containing nano-hydroxyapatite (nHA) following treatment with different organic acids at target pH values of 3.5 and 5.0. The horizontal line indicates the stoichiometric mass-based Ca:P ratio of hydroxyapatite (Ca:PHA = 2.16). Bars represent means, and error bars indicate ±SD of three technical incubation replicates obtained from one independently prepared suspension per OA × pH combination. Results were evaluated descriptively, and no inferential comparisons were performed.

The OA follow-up experiment supports a contribution of OAs to nHA mobilization under the applied in vitro conditions. All tested OAs resulted in measurable P concentrations in the supernatant fraction, and OA-mediated adjustment to pH 3.5 was descriptively associated with higher observed supernatant P concentrations than adjustment to pH 5.0, consistent with an influence of proton activity on HA dissolution [53]. However, the amounts of acid required to reach the respective target pH differed markedly among OAs. PSE should therefore be interpreted descriptively rather than as a definitive measure of intrinsic OA efficiency. The observation of some OAs being more efficient in lowering the pH may be particularly relevant under soil conditions, where root- and microbially derived OAs are released only locally and in limited amounts and are rapidly buffered or degraded. Although the target pH was identical within each pH treatment, supernatant P concentrations and Ca:P ratios differed among OAs, indicating that proton activity alone is unlikely to explain the observed mobilization patterns. These descriptive differences are consistent with additional OA-specific interactions with Ca, including complexation. However, the present design does not allow the relative contributions of proton activity and Ca interactions to be quantified independently. The comparatively low Ca:P ratio observed for oxalate is consistent with Ca complexation and possible calcium oxalate precipitation (Figure A5). Structural properties of citrate, lactate, and gluconate may likewise facilitate interactions with Ca^2+^ [29,66,67].

The production of different OAs by PSB may reflect ecological and metabolic trade-offs. From a microbial perspective, gluconic acid may additionally be metabolically advantageous because its production can proceed directly through glucose oxidation and may require a lower metabolic investment than citrate synthesis, which is more closely linked to central metabolic pathways such as the tricarboxylic acid cycle [68,69]. As P mobilization primarily supports microbial nutrient acquisition, moderate solubilization efficiencies might be sufficient for PSB fitness. At the same time, strong acidification caused by OA release may inhibit bacterial growth and favor more pH-tolerant competitors in soil ecosystems [70]. Thus, PSB-mediated OA production likely reflects a balance between solubilization efficiency, metabolic costs, and microbial competitiveness. However, OA-mediated dissolution alone may not fully explain the high solubilization performance of PSB. In some cases, bacterial cultures achieved higher dissolution levels than OAs applied at high concentrations. Additional mechanisms, including extracellular polymeric substances-mediated biofilm formation on the nanoparticle surface, could further enhance P release by promoting local accumulation of solubilizing metabolites, providing binding sites for Ca^2+^, involving exoenzymes, and sustaining diffusion processes [71,72]. Overall, these findings support hypothesis (i): PSB substantially increased P concentrations in the supernatant fraction of nHA-containing cultures, with OAs potentially contributing to, but not fully explaining, the observed mobilization patterns.

### 4.2. Effects of nHA and PSB on Soil P Availability

To evaluate hypothesis (ii), soil P availability was assessed using CAT extraction. P supply level was the dominant factor controlling CAT-extractable soil P, leading to strongly differentiated responses between low and moderate P supply. Despite the substantially greater amounts of CAT-extractable soil P under moderate P supply, this increase was not fully reflected in plant P uptake and DM production at harvest. Under low P supply, the results were consistent and showed low variability. nHA increased CAT-extractable soil P across all treatments, with +nHA+B reaching the highest values (Figure 4c). Thus, the outcome generally aligned with the hypothesis; however, absolute values remained low, limiting agronomic relevance, and the effects were smaller than PSB-induced increases in soil P availability reported in other studies [73,74]. Under moderate P supply, the effects were less consistent and variability increased. nHA did not consistently enhance CAT-extractable soil P, whereas +nHA+sM showed the highest values (Figure 4c). This does not necessarily imply enhanced P mobilization, as the comparatively lower DM production in this treatment may have resulted in greater residual P remaining in the soil. No additional increase in CAT-extractable soil P attributable to PSB was observed under moderate P supply; overall, treatment effects became less distinct with increasing P supply.

The comparatively low levels of CAT-extractable soil P despite the strong in vitro mobilization may, in part, be explained by limited mobilization from nHA. Under the given conditions, PSB could have been less effective than under in vitro conditions. While severe P deficiency could intensify plant–microbe interactions, for example through increased root exudation [75], the measured bulk soil pH remained high at approximately 7.2 across all treatments. This indicates a strong buffering capacity of the soil. However, CAT-extractable P was determined in bulk soil collected after harvest, and this operational measure does not resolve localized P dynamics within the fertilizer depot or rhizosphere [76]. Liming with CaCO_3_ represents a standard agricultural management practice and was physiologically necessary to maintain the soil pH within a physiologically suitable range for the soil ecosystem and to prevent excessive acidification in the pot experiment, but likely constituted a major limitation for PSB-mediated dissolution processes [77]. Strong soil buffering likely rapidly neutralized protons released by OAs, thereby limiting proton-driven dissolution and maintaining conditions favoring low nHA solubility and potential re-precipitation of mobilized P at high pH [78]. Elevated Ca^2+^ concentrations may additionally have occupied OA complexation sites, thus reducing Ca-mediated dissolution mechanisms. The moderate temperature likely constrained microbial activity, as the applied PSB have temperature optima of approximately 28 °C, whereas only 18/14 °C prevailed in the pot experiment, and enzymatic soil processes are considerably slower at lower temperatures [38,39].

Furthermore, microbial P immobilization may have contributed to the observed P dynamics. Figure 1 shows lower observed P concentrations at the later sampling time points for several strains; however, because no inferential comparisons among sampling days were performed, this temporal pattern should be interpreted descriptively. The pattern is compatible with microbial P uptake and immobilization [79], although other processes such as re-precipitation cannot be excluded. Microbial P immobilization can be further promoted under conditions of high nitrogen and carbon availability [80], particularly in the nHA depot treatments and through the addition of sterile medium and PSB. Because CAT extraction does not quantify P temporarily immobilized in microbial biomass, this pool would not be represented in the CAT-extractable P fraction. Localized P solubilization and microbial P immobilization may therefore have occurred simultaneously. In addition to microbial immobilization, P diffusing out of the extracellular polymeric substances matrix could be re-precipitated under conditions of pH 7.2 and high Ca^2+^ availability due to liming [78]. Consequently, P may be solubilized from nHA without being immediately detectable in the CAT extraction. Overall, hypothesis (ii) was only partially supported. Under low P supply, the combined nHA+PSB treatment showed the highest CAT-extractable P and the numerically highest shoot P uptake. However, shoot P uptake did not differ significantly among treatments, and the combined treatment did not consistently outperform the corresponding nHA and sterile-medium treatments across both response variables. An additional PSB-mediated benefit could therefore not be demonstrated unequivocally. Under moderate P supply, living PSB did not provide a consistent benefit relative to the corresponding sterile-medium control.

### 4.3. Effects of nHA and PSB on P Uptake and Plant Growth

To evaluate hypothesis (iii), the results first show that the higher P supply in the moderate fertilization treatment led to increased P uptake and DM production irrespective of treatment, confirming the high effectiveness of mineral fertilization with Na_2_HPO_4_. Plant P concentrations were nevertheless relatively similar between fertilization levels and were in some cases higher in treatments with lower DM, indicating a dilution effect under more vigorous growth [81]. Under low P supply, shoot P uptake did not differ significantly among treatments (Figure 4b), although +nHA+B showed the numerically highest value. All PREnHA estimates remained below 1%, indicating a low apparent treatment-associated recovery relative to P applied as nHA under severe deficiency (Figure 5a). The PRE_B_ point estimate was 2.8%, representing a small positive contrast between +nHA+B and +nHA+sM (Figure 5b). Under moderate P supply, variability in P uptake was high, which may indicate heterogeneous root access to the locally placed nHA. As P mobility in the soil solution is low, the root must physically access the fertilizer depot for plant uptake to occur [82]. Under moderate P supply, the difference in shoot P uptake between the nHA treatment and its corresponding non-nHA control was equivalent to an apparent shoot P recovery of 6.6% of the P applied as nHA (Figure 5a). This difference-based estimate does not provide source-specific evidence that the additionally acquired P originated directly from nHA. Nevertheless, plant-mediated mobilization in the rhizosphere, potentially involving the release of organic acids and other root exudates, may have contributed to the observed treatment difference [83,84]. Previous studies further reveal that nHA particles may partially penetrate root tissues via apoplastic pathways before dissolving and releasing orthophosphate within the root [85]. Under conditions of high buffering capacity, such direct rhizosphere mobilization could be advantageous, as spatial transport of dissolved P is limited at high pH and elevated Ca^2+^ availability, where P is prone to re-precipitation [78]. Under moderate P supply, plants could also increase the exudation of OAs [86], whereas severe P deficiency can constrain metabolism to a degree that limits this adaptive response [87,88].

In contrast, the addition of PSB or their sterile medium did not consistently improve P uptake and in some cases even reduced it, which may partly have reflected enhanced microbial uptake and immobilization of available P relative to plant acquisition [89]. If microbial immobilization occurred, plants would have competed with the soil microbial community for available P, with immobilized P becoming potentially available again only after microbial turnover and remineralization. This process is, under sufficient moisture conditions, time- and temperature-dependent, and the duration of 51 days after planting under moderate temperatures may have been insufficient for positive effects to emerge [89]. Accordingly, potential benefits of microbially immobilized P may become more apparent only over longer growth periods that allow sufficient microbial turnover and remineralization to occur. In addition, the plant’s capacity for P acquisition was likely constrained by high pH, resulting in reduced proton availability for proton-coupled phosphate uptake and temperature-related reductions in P uptake capacity [62,90]. PRE_B_ point estimates were positive under both P supply levels, but the magnitude of the contrasts remained limited (Figure 5b). These limited responses may have been influenced by temperature, experimental duration, and soil buffering capacity.

Plant growth was primarily determined by the level of P supply. The higher R:S under P deficiency (Figure 3) corresponds to a typical adaptation to P limitation [91]. Under low P supply, no effects of nHA or PSB on DM were observed. Under moderate P supply, the nHA-containing depot treatment without added medium or bacterial inoculum produced the highest shoot DM, whereas +sM and +B resulted in lower shoot DM than the control (Figure 3). This pattern closely mirrored the treatment responses observed for shoot P uptake (Figure 4b). However, because sucrose was co-applied with nHA in all nHA-containing treatments, the higher shoot P uptake and biomass observed in the +nHA treatment cannot be attributed to nHA alone. Microbial P immobilization may have contributed to the lower responses in treatments receiving sterile medium or living PSB. However, because both sucrose and medium components could stimulate microbial activity, these effects cannot be assigned specifically to the inoculated PSB [80]. This may be particularly relevant for maize, whose early establishment and growth strongly depend on sufficient P availability during initial developmental stages [40,90]. Overall, the live consortium did not provide a consistent additional benefit relative to the corresponding sterile-medium control. Thus, hypothesis (iii) was not supported with respect to an additional PSB effect, whereas the apparent benefit of the nHA–sucrose depot formulation requires confirmation in an experimental design that separates the effects of both components.

### 4.4. Limitations of Pot Experiments and Transferability of In Vitro Findings

The limitations of the experiment might explain why the in vitro effects were not fully transferable to the pot experiment. Localized P fertilizer depots may not be reached by roots within the experimental period, potentially limiting efficient exploitation of nHA by PSB compared with a more homogeneous distribution. Liming was agronomically justified but may have reduced both biological and chemical P-solubilization processes. Strong soil buffering likely constrained proton-driven dissolution, while elevated Ca^2+^ availability may have further reduced Ca-complexation effects of OAs. The addition of sucrose may have stimulated microbial activity not only of the inoculated PSB but also of indigenous microbial communities, potentially promoting microbial P immobilization and thereby reducing the immediate detectability of mobilized P in CAT extracts or plant uptake. Because sucrose was included only in the nHA-containing treatments, contrasts between treatments with and without nHA represent the combined effect of the nHA depot fertilizer formulation and sucrose addition rather than an isolated nHA effect. Subsequent P release through microbial turnover may have occurred asynchronously with plant demand, particularly in maize, while reduced temperatures likely further slowed microbial turnover and remineralization. The survival, spatial distribution, and activity of the inoculated strains were not directly assessed. Therefore, the limited plant response cannot be attributed specifically to insufficient P-solubilizing activity, poor establishment of the inoculants, or inadequate spatial contact between the bacteria and the nHA fertilizer depots. Moreover, the pot experiment evaluated only the four-strain consortium and did not include single-strain inoculations. Consequently, the soil experiment does not allow treatment responses to be attributed to individual strains, nor does it permit assessment of whether the strongest in vitro performers retained their activity in soil or whether synergistic or antagonistic interactions occurred among consortium members. Future experiments should therefore compare selected individual strains with defined consortia under identical soil conditions to determine whether consortium formation provides an advantage over single-strain inoculation.

The influence of temperature on the solubilization capacity and survival of individual strains could be assessed during future in vitro strain-selection experiments. Among the potential constraints, soil chemical buffering can be identified with greater confidence under the present experimental conditions because the consistently high soil pH and liming directly indicate conditions unfavorable for proton-driven nHA dissolution. In contrast, insufficient inoculum establishment and inadequate spatial contact among roots, PSB, and the nHA fertilizer depots remain plausible but unverified because colonization and root access were not directly assessed. Moderate temperature and microbial P immobilization may also have contributed, but their individual effects could likewise not be isolated experimentally. Future research should therefore aim to identify the key variables limiting the transfer from simplified in vitro systems to more complex soil environments, including interactions with other microorganisms such as arbuscular mycorrhizal fungi and the potential effects of combining them with PSB. However, any modifications must remain agronomically realistic and avoid creating conditions that are not representative of field systems. The long-term objective should be to translate the solubilization effects observed in vitro into effective and reliable applications under open-field conditions.

## 5. Conclusions

The selected bacterial strains increased P and Ca concentrations in the supernatant fraction of nHA-containing cultures under in vitro conditions. In the soil-based pot experiment, however, the live consortium did not provide a statistically consistent benefit relative to the corresponding sterile-medium controls. Under low P supply, the combined nHA+PSB treatment showed the highest CAT-extractable P and the numerically highest shoot P uptake, although shoot P uptake did not differ significantly among treatments and no clear additional benefit of combining nHA with PSB was demonstrated. Under moderate P supply, the nHA-containing depot treatment without added medium or bacterial inoculation produced the highest shoot P uptake and biomass, although the effects of nHA and sucrose could not be separated. These findings show that effects observed in simplified in vitro systems cannot be transferred directly to buffered soil conditions. Synthetic nHA should therefore be regarded here as a model material for poorly soluble calcium phosphate phases rather than as a tested recycled P fertilizer. Future research should focus on management strategies and environmental conditions that improve the transferability of microbial P mobilization from controlled systems to field-relevant conditions, including optimization of fertilizer–inoculant placement and evaluation across soils differing in buffering capacity and under different temperature regimes, as well as the potential role of complementary microbial partners such as arbuscular mycorrhizal fungi.

## Figures and Tables

**Table 1:** Bacterial strains used in the experiment, including DSMZ (German Collection of Microorganisms and Cell Cultures, Braunschweig, Germany) accession numbers, DSMZ media, and corresponding cell densities expressed as colony-forming units (CFU mL^−1^), and references indicating phosphate-solubilizing properties. *Paraburkholderia terricola* (DSM 17221) did not form colonies on the CFU counting plates, and therefore no CFU could be determined (“n.d.”). Literature on phosphorus solubilizing capacity is included under references.

Strain Name	DSMZ Number	DSMZ Medium	CFU (OD_600_ = 0.1)	References
*Bacillus subtilis*	DSM 21393	1 + MnSO_4_	5.09 × 10^7^	[45,46]
*Pseudomonas alloputida*	DSM 6125	1 + MnSO_4_	1.38 × 10^8^	[47]
*Variovorax paradoxus*	DSM 30034	1 + MnSO_4_	2.78 × 10^8^	[48]
*Pseudomonas graminis*	DSM 100941	92	2.82 × 10^7^	[49,50]
*Pseudomonas rhodesiae*	DSM 7527	220	2.28 × 10^8^	[20,51]
*Paraburkholderia terricola*	DSM 17221	220	n.d.	[20,52]

**Table 2:** Qualitative organic acid (OA) profiling of selected PSB strains by nuclear magnetic resonance. Culture supernatants were analysed for OAs, including succinate, ascorbate, acetate, citrate, and lactate (mmol L^−1^). Due to the exploratory design without biological replication, the dataset is not statistically robust and is intended as a qualitative screening (*n* = 1). “n.d.” indicates not detected.

Strain	Succinate	Ascorbate	Acetate	Citrate	Lactate
DSM 6125	0.001	0.105	0.028	0.105	n.d.
DSM 100941	0.002	0.075	0.307	n.d.	0.168
DSM 7527	0.122	0.112	1.611	n.d.	n.d.
DSM 17221	0.004	0.034	0.139	n.d.	0.013

**Table 3:** Nutrient composition of the soil in the pot experiment, including initial contents (mg kg^−1^ soil), applied fertilizer forms, total nutrient additions (mg kg^−1^ soil, mg pot^−1^), phosphorus treatments (mineral fertilizer and nHA) and lime application based on soil pH (mg pot^−1^). P amounts were summed irrespective of differences in availability among P forms. Values reported for nutrient additions and total nutrient contents refer to the amount of the respective nutrient indicated in the ‘Nutrient’ column, not to the mass of the fertilizer compound. For CaCO_3_, the reported amount refers to the mass of the compound.

Nutrient		Initial Content	Form	Nutrient Additions	Total	Total Pot
		mg kg^−1^ soil		mg kg^−1^ soil	mg kg^−1^ soil	mg pot^−1^
Magnesium		76.3 ^1^	MgSO_4_ · 7H_2_O	43.7	120.0	299.9
Potassium		63.5 ^1^	K_2_SO_4_	146.5	210.0	525.0
Ammonium-N (NH_4_^+^-N)		1.7 ^2^	NH_4_NO_3_	88.0	89.7	224.4
Nitrate-N (NO_3_^−^-N)		5.2 ^2^	Ca(NO_3_)_2_ · 4H_2_O	266.0	271.2	678.0
Phosphorus	low	10.7 ^1^	-	-	10.7	26.7
	low+nHA	10.7 ^1^	nHA	14.8	25.5	63.7
	moderate	10.7 ^1^	Na_2_HPO_4_ · 2H_2_O	29.3	40.0	100.0
	moderate+nHA	10.7 ^1^	Na_2_HPO_4_ · 2H_2_O +nHA	44.1	54.8	137.0
Lime		pH 5.72 ^2^	CaCO_3_	2200	2200	5500

^1^ was analyzed by LUFA NordWest; ^2^ was analyzed in our labs.

## Data Availability

The data supporting the findings of this study are available from the corresponding author upon reasonable request.

## Appendix A

**Table A1:** Final concentrations of the organic acids (mmol L^−1^) after addition of 0.5 M organic acid solutions to phosphorus-free medium containing nano-hydroxyapatite (nHA) to adjust the suspensions to pH 3.5 and pH 5.0. Concentrations used for PSE calculations.

Organic Acid	pH 5.0	pH 3.5
Ascorbic acid	0.83	38.46
Succinic acid	1.00	20.92
Acetic acid	1.99	56.21
Lactic acid	3.31	12.99
Oxalic acid	1.00	2.98
Propionic acid	1.33	77.46
Citric acid	0.67	4.79
Gluconic acid	2.49	16.13

## References

[1] Islam M.M., Rengel Z., Storer P., Siddique K.H.M., Solaiman Z.M. (2023). Phosphorus Fertilisation Differentially Influences Growth, Morpho-Physiological Adaptations and Nutrient Uptake of Industrial Hemp (*Cannabis Sativa* L.). Plant Soil.

[2] Johnston A.E., Poulton P.R. (2019). Phosphorus in Agriculture: A Review of Results from 175 Years of Research at Rothamsted, UK. J. Environ. Qual..

[3] Calderón-Vázquez C., Sawers R.J.H., Herrera-Estrella L. (2011). Phosphate Deprivation in Maize: Genetics and Genomics. Plant Physiol..

[4] Ji L., Tian C., Kuramae E.E. (2023). Phosphorus-Mediated Succession of Microbial Nitrogen, Carbon, and Sulfur Functions in Rice-Driven Saline-Alkali Soil Remediation. Soil Biol. Biochem..

[5] Dick C.F., Dos-Santos A.L.A., Meyer-Fernandes J.R. (2011). Inorganic Phosphate as an Important Regulator of Phosphatases. Enzym. Res..

[6] Rengel Z., Cakmak I., White P.J. (2023). Marschner’s Mineral Nutrition of Plants.

[7] Tiessen H., White P.J., Hammond J.P. (2008). Phosphorus in the Global Environment. The Ecophysiology of Plant-Phosphorus Interactions.

[8] Hedley M., McLaughlin M., Sims T., Sharpley A.N. (2005). Reactions of Phosphate Fertilizers and By-Products in Soils. Agronomy Monographs.

[9] Von Wandruszka R. (2006). Phosphorus Retention in Calcareous Soils and the Effect of Organic Matter on Its Mobility. Geochem. Trans..

[10] Withers P.J.A., Sylvester-Bradley R., Jones D.L., Healey J.R., Talboys P.J. (2014). Feed the Crop Not the Soil: Rethinking Phosphorus Management in the Food Chain. Environ. Sci. Technol..

[11] Carpenter S.R., Caraco N.F., Correll D.L., Howarth R.W., Sharpley A.N., Smith V.H. (1998). Nonpoint Pollution of Surface Waters with Phosphorus and Nitrogen. Ecol. Appl..

[12] Kang J., Amoozegar A., Hesterberg D., Osmond D.L. (2011). Phosphorus Leaching in a Sandy Soil as Affected by Organic and Inorganic Fertilizer Sources. Geoderma.

[13] Fixen P.E., Johnston A.M. (2012). World Fertilizer Nutrient Reserves: A View to the Future. J. Sci. Food Agric..

[14] Walsh M., Schenk G., Schmidt S. (2023). Realising the Circular Phosphorus Economy Delivers for Sustainable Development Goals. npj Sustain. Agric..

[15] Said H.A., Noukrati H., Mansouri H., Perreault F., Oukarroum A., Youcef H.B. (2026). Performance and Challenges of Hydroxyapatite and Its Nanocomposite-Based Nanofertilizers: A Review. Pedosphere.

[16] Harkat M., Alleg S., Chemam R., Azzaza S., Mokhtari M., Dhahri E. (2021). Synthesis and Characterization of Thermally Stable Hydroxyapatite. Ann. Chim. Sci. Mat..

[17] Barua E., Das A., Pamu D., Deoghare A.B., Deb P., Lala S.D., Chatterjee S. (2019). Effect of Thermal Treatment on the Physico-Chemical Properties of Bioactive Hydroxyapatite Derived from Caprine Bone Bio-Waste. Ceram. Int..

[18] Chen J., Tang S., Yan F., Zhang Z. (2020). Efficient Recovery of Phosphorus in Sewage Sludge through Hydroxylapatite Enhancement Formation Aided by Calcium-Based Additives. Water Res..

[19] Pilotto L., Scalera F., Piccirillo C., Marchiol L., Zuluaga M.Y.A., Pii Y., Cesco S., Civilini M., Fellet G. (2025). Phosphorus Release from Nano-Hydroxyapatite Derived from Biowastes in the Presence of Phosphate-Solubilizing Bacteria: A Soil Column Experiment. J. Agric. Food Chem..

[20] Quattrocelli P., Piccirillo C., Kuramae E.E., Pullar R.C., Ercoli L., Pellegrino E. (2025). Synergistic Interaction of Phosphate Nanoparticles from Fish By-Products and Phosphate-Solubilizing Bacterial Consortium on Maize Growth and Phosphorus Cycling. Sci. Total Environ..

[21] Lehner R. (1940). Untersuchungen über die Wasserlöslichkeit von Hydroxylapatit im Temperaturbereich von 20–350 °C. Ph.D. Thesis.

[22] Montalvo D., McLaughlin M.J., Degryse F. (2015). Efficacy of Hydroxyapatite Nanoparticles as Phosphorus Fertilizer in Andisols and Oxisols. Soil Sci. Soc. Am. J..

[23] Shen J., Yuan L., Zhang J., Li H., Bai Z., Chen X., Zhang W., Zhang F. (2011). Phosphorus Dynamics: From Soil to Plant. Plant Physiol..

[24] European Commission (2011). Commission Recommendation of 18 October 2011 on the Definition of Nanomaterial.

[25] Ditta A., Arshad M. (2016). Applications and Perspectives of Using Nanomaterials for Sustainable Plant Nutrition. Nanotechnol. Rev..

[26] Weeks J.J., Hettiarachchi G.M. (2019). A Review of the Latest in Phosphorus Fertilizer Technology: Possibilities and Pragmatism. J. Environ. Qual..

[27] Paries M., Gutjahr C. (2023). The Good, the Bad, and the Phosphate: Regulation of Beneficial and Detrimental Plant–Microbe Interactions by the Plant Phosphate Status. New Phytol..

[28] Hinsinger P. (2001). Bioavailability of Soil Inorganic P in the Rhizosphere as Affected by Root-Induced Chemical Changes: A Review. Plant Soil.

[29] Farhat M.B., Boukhris I., Chouayekh H. (2015). Mineral Phosphate Solubilization by Streptomyces Sp. CTM396 Involves the Excretion of Gluconic Acid and Is Stimulated by Humic Acids. FEMS Microbiol. Lett..

[30] Iqbal Z., Ahmad M., Raza M.A., Hilger T., Rasche F. (2024). Phosphate-Solubilizing Bacillus Sp. Modulate Soil Exoenzyme Activities and Improve Wheat Growth. Microb. Ecol..

[31] Rawat P., Das S., Shankhdhar D., Shankhdhar S.C. (2021). Phosphate-Solubilizing Microorganisms: Mechanism and Their Role in Phosphate Solubilization and Uptake. J. Soil Sci. Plant Nutr..

[32] Flemming H.-C., Van Hullebusch E.D., Little B.J., Neu T.R., Nielsen P.H., Seviour T., Stoodley P., Wingender J., Wuertz S. (2025). Microbial Extracellular Polymeric Substances in the Environment, Technology and Medicine. Nat. Rev. Microbiol..

[33] Xiao J., Hara A.T., Kim D., Zero D.T., Koo H., Hwang G. (2017). Biofilm Three-Dimensional Architecture Influences in Situ pH Distribution Pattern on the Human Enamel Surface. Int. J. Oral Sci..

[34] Hameeda B., Harini G., Rupela O.P., Wani S.P., Reddy G. (2008). Growth Promotion of Maize by Phosphate-Solubilizing Bacteria Isolated from Composts and Macrofauna. Microbiol. Res..

[35] Park Y., Solhtalab M., Thongsomboon W., Aristilde L. (2022). Strategies of Organic Phosphorus Recycling by Soil Bacteria: Acquisition, Metabolism, and Regulation. Environ. Microbiol. Rep..

[36] Santana C.A., Piccirillo C., Pereira S.I.A., Pullar R.C., Lima S.M., Castro P.M.L. (2019). Employment of Phosphate Solubilising Bacteria on Fish Scales–Turning Food Waste into an Available Phosphorus Source. J. Environ. Chem. Eng..

[37] Elhaissoufi W., Khourchi S., Ibnyasser A., Ghoulam C., Rchiad Z., Zeroual Y., Lyamlouli K., Bargaz A. (2020). Phosphate Solubilizing Rhizobacteria Could Have a Stronger Influence on Wheat Root Traits and Aboveground Physiology Than Rhizosphere P Solubilization. Front. Plant Sci..

[38] Jha A., Saxena J., Sharma V. (2013). Investigation on Phosphate Solubilization Potential of Agricultural Soil Bacteria as Affected by Different Phosphorus Sources, Temperature, Salt, and pH. Commun. Soil Sci. Plant Anal..

[39] Menichetti L., Reyes Ortigoza A.L., García N., Giagnoni L., Nannipieri P., Renella G. (2015). Thermal Sensitivity of Enzyme Activity in Tropical Soils Assessed by the Q10 and Equilibrium Model. Biol. Fertil. Soils.

[40] Plénet D., Etchebest S., Mollier A., Pellerin S. (2000). Growth Analysis of Maize Field Crops under Phosphorus Deficiency. Plant Soil.

[41] Nkebiwe P.M., Weinmann M., Bar-Tal A., Müller T. (2016). Fertilizer Placement to Improve Crop Nutrient Acquisition and Yield: A Review and Meta-Analysis. Field Crops Res..

[42] O’Callaghan M., Ballard R.A., Wright D. (2022). Soil Microbial Inoculants for Sustainable Agriculture: Limitations and Opportunities. Soil Use Manag..

[43] Tennant R., Rutten P. (2019). Calibration Protocol-Conversion of OD600 to Colony Forming Units (CFUs) V1.

[44] Nautiyal C.S. (1999). An Efficient Microbiological Growth Medium for Screening Phosphate Solubilizing Microorganisms. FEMS Microbiol. Lett..

[45] Chea L., Alhussein M., Karlovsky P., Pawelzik E., Naumann M. (2024). Adaptation of Potato Cultivars to Phosphorus Variability and Enhancement of Phosphorus Efficiency by Bacillus Subtilis. BMC Plant Biol..

[46] Murad S., Ahmad M., Hussain A., Ali S., Al-Ansari N., Mattar M.A. (2024). Efficacy of DAP Coated with Bacterial Strains and Their Metabolites for Soil Phosphorus Availability and Maize Growth. Sci. Rep..

[47] Pilotto L., Zuluaga M.Y.A., Scalera F., Piccirillo C., Marchiol L., Civilini M., Pii Y., Cesco S., Fellet G. (2024). Sustainable Crop Fertilization by Combining Biogenic Nano-Hydroxyapatite and P Solubilizing Bacteria: Observations on Barley. Plant Nano Biol..

[48] Zheng B.-X., Ding K., Yang X.-R., Wadaan M.A.M., Hozzein W.N., Peñuelas J., Zhu Y.-G. (2019). Straw Biochar Increases the Abundance of Inorganic Phosphate Solubilizing Bacterial Community for Better Rape (Brassica Napus) Growth and Phosphate Uptake. Sci. Total Environ..

[49] Crovadore J., Calmin G., Chablais R., Cochard B., Schulz T., Lefort F. (2016). Whole-Genome Sequence of Pseudomonas Graminis Strain UASWS1507, a Potential Biological Control Agent and Biofertilizer Isolated in Switzerland. Genome Announc..

[50] Kolytaitė A., Vaitiekūnaitė D., Antanynienė R., Baniulis D., Frercks B. (2022). Monilinia Fructigena Suppressing and Plant Growth Promoting *Endophytic Pseudomonas* Spp. Bacteria Isolated from Plum. Microorganisms.

[51] Rolón-Cárdenas G.A., Arvizu-Gómez J.L., Pacheco-Aguilar J.R., Vázquez-Martínez J., Hernández-Morales A. (2021). Cadmium-Tolerant Endophytic Pseudomonas Rhodesiae Strains Isolated from Typha Latifolia Modify the Root Architecture of Arabidopsis Thaliana Col-0 in Presence and Absence of Cd. Braz. J. Microbiol..

[52] Gasser I., Cardinale M., Müller H., Heller S., Eberl L., Lindenkamp N., Kaddor C., Steinbüchel A., Berg G. (2011). Analysis of the Endophytic Lifestyle and Plant Growth Promotion of Burkholderia Terricola ZR2-12. Plant Soil.

[53] Wang D., Xie Y., Jaisi D.P., Jin Y. (2016). Effects of Low-Molecular-Weight Organic Acids on the Dissolution of Hydroxyapatite Nanoparticles. Environ. Sci. Nano.

[54] Gulati A., Sharma N., Vyas P., Sood S., Rahi P., Pathania V., Prasad R. (2010). Organic Acid Production and Plant Growth Promotion as a Function of Phosphate Solubilization by Acinetobacter Rhizosphaerae Strain BIHB 723 Isolated from the Cold Deserts of the Trans-Himalayas. Arch. Microbiol..

[55] Saeid A., Prochownik E., Dobrowolska-Iwanek J. (2018). Phosphorus Solubilization by Bacillus Species. Molecules.

[56] Stephen J., Jisha M.S. (2011). Gluconic Acid Production as the Principal Mechanism of Mineral Phosphate Solubilization by Burkholderia Sp. (MTCC 8369). J. Trop. Agric..

[57] Thun R., Hoffmann G., Bassler R., VDLUFA (2016). Methodenbuch: Die Untersuchung von Böden.

[58] IUSS Working Group WRB (2022). World Reference Base for Soil Resources: International Soil Classification System for Naming Soils and Creating Legends for Soil Maps.

[59] Landesamt für Bergbau, Energie und Geologie (LBEG) Bodenkarte von Niedersachsen 1:50,000 (BK50N); NIBIS® Kartenserver. https://nibis.lbeg.de/cardomap3/.

[60] LWK Niedersachsen (2026). Empfehlungen 2026-Pflanzenbau Und Pflanzenschutz.

[61] Johnston J., Syers J.K. (2009). A New Approach to Assessing Phosphorus Use Efficiency in Agriculture. Better Crops.

[62] Smith A.N., Posner A.M., Quirk J.P. (1974). Incongruent Dissolution and Surface Complexes of Hydroxyapatite. J. Colloid Interface Sci..

[63] El Habil-Addas F., Aarab S., Rfaki A., Laglaoui A., Bakkali M., Arakrak A. (2017). Screening of Phosphate Solubilizing Bacterial Isolates for Improving Growth of Wheat. Eur. J. Biotechnol. Biosci..

[64] Pande A., Pandey P., Mehra S., Singh M., Kaushik S. (2017). Phenotypic and Genotypic Characterization of Phosphate Solubilizing Bacteria and Their Efficiency on the Growth of Maize. J. Genet. Eng. Biotechnol..

[65] Marra L.M., De Oliveira-Longatti S.M., Soares C.R.F.S., Olivares F.L., Moreira F.M.D.S. (2019). The Amount of Phosphate Solubilization Depends on the Strain, C-Source, Organic Acids and Type of Phosphate. Geomicrobiol. J..

[66] Barrow N.J., Debnath A., Sen A. (2018). Mechanisms by Which Citric Acid Increases Phosphate Availability. Plant Soil.

[67] Chen W., He P., Zhang H., Duan H., Shao L., Lü F. (2023). The Low Dose of L-Lactic Acid as Loess Soil Amendment Enhances Wheat Rhizosphere Microecosystem. Rhizosphere.

[68] Buch A., Archana G., Naresh Kumar G. (2008). Metabolic Channeling of Glucose towards Gluconate in Phosphate-Solubilizing Pseudomonas Aeruginosa P4 under Phosphorus Deficiency. Res. Microbiol..

[69] Rodriguez H., Gonzalez T., Goire I., Bashan Y. (2004). Gluconic Acid Production and Phosphate Solubilization by the Plant Growth-Promoting Bacterium *Azospirillum* Spp.. Naturwissenschaften.

[70] Rousk J., Brookes P.C., Bååth E. (2009). Contrasting Soil pH Effects on Fungal and Bacterial Growth Suggest Functional Redundancy in Carbon Mineralization. Appl. Environ. Microbiol..

[71] Oves M., Khan M.S., Zaidi A. (2013). Chromium Reducing and Plant Growth Promoting Novel Strain Pseudomonas Aeruginosa OSG41 Enhance Chickpea Growth in Chromium Amended Soils. Eur. J. Soil Biol..

[72] Prabhu N., Borkar S., Garg S. (2019). Phosphate Solubilization by Microorganisms. Advances in Biological Science Research.

[73] Sundara B., Natarajan V., Hari K. (2002). Influence of Phosphorus Solubilizing Bacteria on the Changes in Soil Available Phosphorus and Sugarcane and Sugar Yields. Field Crops Res..

[74] Zhang Y., Shi J., Wu J., Ye R., Wang K., Ren J., Fang X., Shi F. (2025). Inoculation with Phosphate-Solubilizing Microorganisms Enhances Soil P Bioavailability, Restructures Microbial Communities, and Promotes Moso Bamboo Growth. Appl. Soil Ecol..

[75] Yang Z., Wu T., Niu J., Zhang L., Chen X., Berndtsson R., Yang T., Dou T. (2025). Phosphorus Stress-Induced Root Exudates Mediated Plant-Microbe Interactions of Melilotus Officinalis. Rhizosphere.

[76] Kreuzeder A., Santner J., Scharsching V., Oburger E., Hoefer C., Hann S., Wenzel W.W. (2018). In Situ Observation of Localized, Sub-Mm Scale Changes of Phosphorus Biogeochemistry in the Rhizosphere. Plant Soil.

[77] Adnan M., Fahad S., Zamin M., Shah S., Mian I.A., Danish S., Zafar-ul-Hye M., Battaglia M.L., Naz R.M.M., Saeed B. (2020). Coupling Phosphate-Solubilizing Bacteria with Phosphorus Supplements Improve Maize Phosphorus Acquisition and Growth under Lime Induced Salinity Stress. Plants.

[78] Tunesi S., Poggi V., Gessa C. (1999). Phosphate Adsorption and Precipitation in Calcareous Soils: The Role of Calcium Ions in Solution and Carbonate Minerals. Nutr. Cycl. Agroecosystems.

[79] Seeling B., Zasoski R.J. (1993). Microbial Effects in Maintaining Organic and Inorganic Solution Phosphorus Concentrations in a Grassland Topsoil. Plant Soil.

[80] Olander L.P., Vitousek P.M. (2004). Biological and Geochemical Sinks for Phosphorus in Soil from a Wet Tropical Forest. Ecosystems.

[81] Jarrell W.M., Beverly R.B. (1981). The Dilution Effect in Plant Nutrition Studies. Advances in Agronomy.

[82] Lambers H., Shane M.W., Cramer M.D., Pearse S.J., Veneklaas E.J. (2006). Root Structure and Functioning for Efficient Acquisition of Phosphorus: Matching Morphological and Physiological Traits. Ann. Bot..

[83] Gaume A., Mächler F., De León C., Narro L., Frossard E. (2001). Low-P Tolerance by Maize (*Zea mays* L.) Genotypes: Significance of Root Growth, and Organic Acids and Acid Phosphatase Root Exudation. Plant Soil.

[84] Ström L. (1997). Root Exudation of Organic Acids: Importance to Nutrient Availability and the Calcifuge and Calcicole Behaviour of Plants. Oikos.

[85] Szameitat A.E., Sharma A., Minutello F., Pinna A., Er-Rafik M., Hansen T.H., Persson D.P., Andersen B., Husted S. (2021). Unravelling the Interactions between Nano-Hydroxyapatite and the Roots of Phosphorus Deficient Barley Plants. Environ. Sci. Nano.

[86] Pantigoso H.A., Yuan J., He Y., Guo Q., Vollmer C., Vivanco J.M. (2020). Role of Root Exudates on Assimilation of Phosphorus in Young and Old Arabidopsis Thaliana Plants. PLoS ONE.

[87] Canarini A., Kaiser C., Merchant A., Richter A., Wanek W. (2019). Root Exudation of Primary Metabolites: Mechanisms and Their Roles in Plant Responses to Environmental Stimuli. Front. Plant Sci..

[88] Carstensen A., Herdean A., Schmidt S.B., Sharma A., Spetea C., Pribil M., Husted S. (2018). The Impacts of Phosphorus Deficiency on the Photosynthetic Electron Transport Chain. Plant Physiol..

[89] Ducousso-Détrez A., Fontaine J., Lounès-Hadj Sahraoui A., Hijri M. (2022). Diversity of Phosphate Chemical Forms in Soils and Their Contributions on Soil Microbial Community Structure Changes. Microorganisms.

[90] Mackay A.D., Barber S.A. (1984). Soil Temperature Effects on Root Growth and Phosphorus Uptake by Corn. Soil Sci. Soc. Am. J..

[91] Fredeen A.L., Rao I.M., Terry N. (1989). Influence of Phosphorus Nutrition on Growth and Carbon Partitioning in *Glycine Max*. Plant Physiol..

